# A scaffolded approach to unearth potential antibacterial components from epicarp of Malaysian *Nephelium lappaceum* L.

**DOI:** 10.1038/s41598-021-92622-0

**Published:** 2021-07-05

**Authors:** Ali Asghar, Yong Chiang Tan, Mohammad Zahoor, Syafiq Asnawi Zainal Abidin, Yoon-Yen Yow, Ezzat Khan, Chandrajit Lahiri

**Affiliations:** 1 Department of Biological Sciences, Sunway University, Petaling Jaya, Malaysia; 2 Department of Biochemistry, University of Malakand, Chakdara, Pakistan; 3 Jeffrey Cheah School of Medicine and Health Science, Monash University Malaysia, Petaling Jaya, Malaysia; 4 Department of Chemistry, University of Bahrain, Sakhir, Bahrain

**Keywords:** Computational biology and bioinformatics, Drug discovery, Microbiology

## Abstract

The emergence and spread of antimicrobial resistance have been of serious concern to human health and the management of bacterial infectious diseases. Effective treatment of these diseases requires the development of novel therapeutics, preferably free of side effects. In this regard, natural products are frequently conceived to be potential alternative sources for novel antibacterial compounds. Herein, we have evaluated the antibacterial activity of the epicarp extracts of the Malaysian cultivar of yellow rambutan fruit (*Nephelium lappaceum L.*) against six pathogens namely, *Bacillus subtilis*, methicillin-resistant *Staphylococcus aureus* (MRSA), *Streptococcus pyogenes, Pseudomonas aeruginosa, Klebsiella pneumoniae* and *Salmonella enterica*. Among a series of solvent extracts, fractions of ethyl acetate and acetone have revealed significant activity towards all tested strains. Chemical profiling of these fractions, via HPLC, LC–MS and GC–MS, has generated a library of potentially bioactive compounds. Downstream virtual screening, pharmacological prediction, and receptor-ligand molecular dynamics simulation have eventually unveiled novel potential antibacterial compounds, which can be extracted for medicinal use. We report compounds like catechin, eplerenone and oritin-4-beta-ol to be computationally inhibiting the ATP-binding domain of the chaperone, DnaK of *P. aeruginosa* and MRSA. Thus, our work follows the objective to propose new antimicrobials capable of perforating the barrier of resistance posed by both the gram positives and the negatives.

## Introduction

Antimicrobial resistance (AMR) has been projected as one of the serious global concerns with issues in the management of infectious diseases caused by Multidrug-Resistant (MDR) bacterial pathogens^1^. These pathogens have been reported to cause 700,000 deaths each year and is estimated to cross over 10 million by 2050^2^. Such alarming rise can be attributed mostly to the prevalent misappropriation of antibiotics in human healthcare systems^3^. This is delineated by the overuse and poor appropriate prescriptions coupled with the lack of new drug development, which have reduced the efficiency of antibiotics as the resistant strains rapidly increases^4^. This necessitates the urgency for unearthing alternate natural therapeutics focused on natural products to be exploited as repertoires of natural bioactive compounds, preferably devoid of side effects and hence, the potential for future drug development.

Natural products, frequently discovered with potent antimicrobial potential, have been used as alternatives with the hope of eliminating the use of synthetic antibiotics, for a long time^5^. Besides serving a wide range of secondary plant metabolites, for instance, alkaloids, tannins, flavonoids and phenolic compounds, with remarkable antimicrobial effects^6^, many plant products have been reported for their antimicrobial potentials, namely, citrus peel^7^, grape seed^8^, cranberry pomace^9^, pomegranate peel^10^ and passion fruit seed^11^. In this regard, the fruit rambutan (*Nephelium lappaceum L*.) has gained attention due to its vast range of bioactive constituents including, but not limited to, vitamin C, vitamin E, carotenes, xanthophylls, tannins and phenolic compounds like geraniin, ellagic acid, quercetin, corilagin and rutin^12^.

While the broad range of biological activities of rambutan includes anticancer, antiviral, antidiabetic and anti-hypercholesterolemic properties^13,14^, few researchers have even reported its in vitro antibacterial activities. For instance, Malini & Maheshkumar^15^ have disclosed significant antibacterial activity of rambutan fruit sap extracts towards *Pseudomonas aeruginosa* while Bhat and Al-daihan^16^ revealed antibacterial activities of its seeds extracts against *Staphylococcus aureus, Streptococcus pyogenes, Bacillus subtilis, Escherichia coli* and *P. aeruginosa.* Moreover, the antibacterial potential of rambutan peel extracts have also been reported against *Vibrio cholerae, Enterococcus faecalis, S. aureus* and *Staphylococcus epidermidis*^17^*.* Furthermore, Sekar et al*.*^18^ comparatively evaluated the antibacterial potency of red and yellow rambutan fruit peels against *S. aureus and S. pyogenes,* to reveal better efficacy of the latter extracts against the tested pathogens. However, a deeper exploration of the potential extracts to unveil new antibacterial compounds have hardly been focused.

In this study, thus, we have delineated a stepwise approach of determining the efficacy of the crude extracts of the epicarp of yellow Malaysian rambutan against clinically important MDR bacterial pathogens e.g., *B. subtilis*, methicillin-resistant *S. aureus* (MRSA), *S. pyogenes, P. aeruginosa, K. pneumoniae* and *S. enterica*. To this end, through HPLC, LC–MS and GC–MS analyses, we have prepared chemical profiling of the ethyl acetate and acetone fractions of the crude extracts with promising antibacterial activities. This revealed their potential chemical determinants which were screened virtually to pharmacologically unveil novel bioactive compounds in parallel to molecular dynamics simulation. This enabled us to elect compounds like catechin, eplerenone and oritin-4-beta-ol, which computationally inhibit the important chaperone protein, DnaK of *P. aeruginosa* and MRSA. Essentially, this report serves to unveil these compounds as novel alternatives to cope with the multidrug-resistant gram-positive and -negative pathogens.

## Results

### Variable antibacterial activity of crude extracts by disc diffusion

We have preliminarily screened the antibacterial activities of the yellow-variety Malaysian Rambutan epicarp crude extracts through disc diffusion assay. We have only used freshly prepared solutions of crude extracts for all the tested pathogens (TP). During such trials, the solvent control (SC), DMSO, did not exercise antibacterial activity against the TP, as manifested by a no inhibition zone. Moreover, none of the sequential extracts exhibited activity against the TP (Table 1), while in the case of direct extracts, the picture was different. Extracts of ethyl acetate (EA) displayed visible activity against MRSA, *B. subtilis* and *S. enterica* but did not show activity against the rest of the TP (RTP). Again, acetone (AC) extracts exhibited markedly prominent activity against *B. subtilis* along with visible activity against *S. pyogenes* and *P. aeruginosa*, which was not observed among the RTP (Table S1).

**Table 1:** Antibacterial activity of *the N. lappaceum* sequential and direct crude extracts via disc diffusion.

Zones of inhibition (mm)										
Species	PC	SC	CF	EA	AC	ET	MT	WT	EA(D)	AC(D)
*B. subtilis*	30.00 ± 0.70	–	–	–	–	–	–	–	8.80 ± 0.65	9.39 ± 0.43
MRSA		–	–	–	–	–	–	–	8.58 ± 0.29	–
*S. pyogenes*		–	–	–	–	–	–	–	–	8.35 ± 0.45
*P. aeruginosa*		–	–	–	–	–	–	–	–	8.00 ± 0.01
*K. pneumoniae*		–	–	–	–	–	–	–	–	–
*S. enterica*		–	–	–	–	–	–	–	6.60 ± 0.28	–

“–” no activity, PC: positive control (Gentamicin 10 µg), SC: solvent control (DMSO < 1%), CF: chloroform, EA: ethyl acetate, AC: acetone, ET: ethanol, MT: methanol, WT: water, all these are sequential. EA(D): ethyl acetate direct; AC(D): acetone direct. The data is expressed as the mean ± standard error of two independent experiments performed in technical triplicates.

### Antibacterial screening of crude extracts via Broth dilution

Next, we have used the broth dilution method for evaluating the antibacterial potential of yellow fruit epicarp crude extracts and calculating the viability percentage of every TP (Table 2). This, in turn, helped us to illustrate the percentages of antibacterial potential of EA and AC fractions from the sequential & direct extracts, against the TP, at a concentration of 250 µg/ml. For the sequential extracts, the fraction from AC exhibited the highest percentage (90) of antibacterial activity against *P. aeruginosa* followed by 71% against MRSA while that from EA showed a 59% effect against *S. pyogenes*. No significant results were observed either against the RTP (Fig. S3A–F) or for the remaining solvent extracts namely, chloroform (CF), ethanol (ET), methanol (MT) and aqueous (water, WT) extracts against all TP (Table S2). In the case of direct extracts with the six solvents used, fractions of EA exhibited inhibition of 80% against *B. subtilis* along with 60, 62, 73 and 72% for MRSA, *P. aeruginosa, S. enterica* and *K. pneumonia,* respectively, without any positive results against *S. pyogenes*. Notably, all tested pathogens were inhibited by AC fractions and the percentage of antibacterial effects were 75, 90, 70, 70, 60 and 75, respectively for MRSA, *B. subtilis, S. pyogenes*, *P. aeruginosa, S. enterica* and *K. pneumonia* (Fig. S1A–D).

**Table 2:** Screening of antibacterial effect of sequential and direct extracts by broth dilution method.

% Inhibition of all tested pathogen						
Microorganisms	S.C	P.C	EA(S)	EA(D)	AC(S)	AC(D)
*B. subtilis*	02 ± 0.00	100 ± 0.00	–	80 ± 1.98	–	90 ± 1.42
MRSA	02	100	–	60 ± 1.49	71 ± 1.91	75 ± 2.04
*S. pyogenes*	02	100	59 ± 1.33	–	–	70 ± 1.40
*P. aeruginosa*	02	100	–	62 ± 1.83	90 ± 1.58	70 ± 2.02
*K. pneumonia*	02	100	–	72 ± 1.79	–	75 ± 2.61
*S. enterica*	02	100	–	73 ± 1.84	–	60 ± 2.74

EA(S): ethyl acetate sequential; AC(S): acetone sequential; EA(D): ethyl acetate direct; AC(D): acetone direct; S.C: solvent control: DMSO (< 1%), P.C: positive control (Gentamicin 10 µg); – : Low activity (less than 50%). The data is expressed as the mean ± standard error of two independent experiments performed in technical triplicates.

The results of EA and AC fractions portrayed notable antibacterial efficiency. We have, thus, utilized them for the identification of bioactive compounds via HPLC, LC–MS and GC–MS analyses.

### Revelation of antioxidants from crude extracts using HPLC–UV

At first, we have conducted HPLC–UV, for preliminary identification of the basic antioxidants present in the Malaysian yellow-rambutan epicarp extracts. All compounds with known antioxidant capacities were identified in comparison with standard phenolic compounds. The identified compounds and their quantification, along with their specific peak position and retention time (Rt) in the chromatogram, are shown in Table 3 and Fig. S2. We identified only three compounds in the EA extract namely, malic acid, vitamin C and chlorogenic acid along with three more in the AC extract. These are epigallocatechin gallate, catechin hydrate and quercetin.

**Table 3:** Identified compounds in *N. lappaceum* ethyl acetate and acetone sequential fractions using HPLC–UV.

Sample extract	RT (min)	Identified compounds	Peak area
EA (S)	12.9	Malic acid	40.441
	4.8	Vitamin C	1717.201
	6.2	Chlorogenic acid	190.156
AC (S)	2.9	Malic acid	4.938
	4.8	Vitamin C	1226.316
	6.2	Chlorogenic acid	134.587
	8.8	Epigallocatechin gallate	205.717
	10.7	Quercetin	85.112
	20.7	Catechin hydrate	44.169

EA (S): Ethyl acetate sequential fraction; AC (S): Acetone sequential fraction.

### Exploration of other chemical determinants through Liquid Chromatography–Mass Spectrometry (LC–MS) Analysis

For a fast, mass-directed exploration of the compounds possibly present in the rambutan epicarp, we have subjected the EA and AC sequential crude extracts to LC–MS analysis (Figs. S3 & S4). Our analysis revealed the presence of 54 and 44 compounds, respectively, in the above-mentioned EA and AC extracts (Tables S3 & S4). We have matched them with the identity of known molecules on the Metlin database, keeping a threshold of Molecular Formula Generator (MFG) scores above 86% along with a ± 2% difference. We further explored the compounds above the mentioned cut-off for their biological activities and carried forward for virtual screening. Notably, 31 compounds from both sets of EA and AC extracts have not been reported to date with any antibacterial activities (Tables S3 & S4).

### Identification of volatile constituents by Gas chromatography–mass spectrometry (GC–MS)

**Figure 1:**
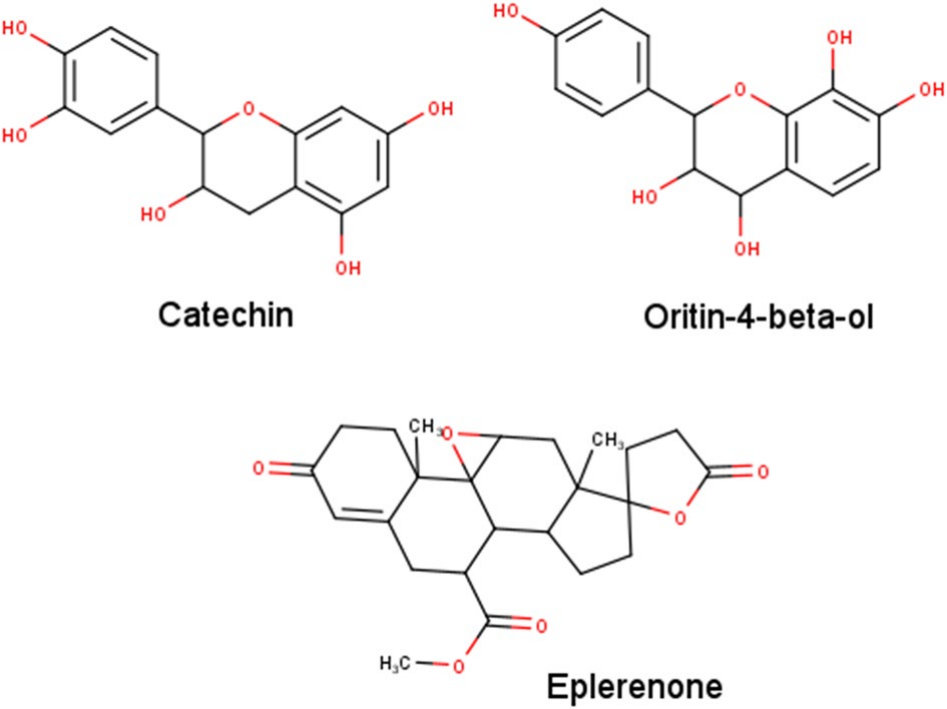
2D Chemical Structures of Catechin, Oritin-4-beta-ol, and Eplerenone generated using MarvinSketch version 20.16 (http://www.chemaxon.com/)^31^.

Hereafter, to identify any volatile organic compounds, present in the EA and AC extracts of Malaysian yellow-variety *N. lappaceum* epicarp, we have exposed them for GC–MS analysis (Fig. S5a–d). Most of the compounds from EA and AC fractions, extracted directly, have been reported with antibacterial activities (Table 4). On the contrary, the compounds of these fractions from sequential extraction have not been reported for any such activity. The chromatogram of these compounds showed mentionable area % scores (above 0.5%) for 3-Methyl-1,2-diazirine (compound 1) and Card-20(22)-enolide, 3-[(6-deoxy-3,4-O-methylenehexopyranos-2-ulos-l-yl) oxy]-5,11/14-trihydroxy-12 -oxo-, (3-beta, 5-alpha, 11-alpha) (compound 2) in the EA extract while, the AC extract showed the presence of Silane, [[(3alpha,5beta,20S)-pregn-11-ene-3,11,17,20-tetrayl] tetrakis(oxy)] tetrakis [trimethyl] and 2,2-Bis[4-[(4,6-dichloro-1,3,5-triazin-2-yl) oxy] phenyl]-1,1,1,3,3,3-hexafluoropropane (Table 4). Of these, compound 2 is known as Eplerenone (Fig. 1) and was found to be an important one in the upcoming analyses.

**Table 4:** Compounds existing in *N. lappaceum* ethyl acetate and acetone (sequential & direct) extract identified by GC–MS analysis.

No	Extracts	Identified compounds	Molecular Formula	R.T. (Min)	Area %	Antibacterial activity report
1	EA (S)	3-Methyl-1,2-diazirine	C_2_H_4_N_2_	3.02	0.776	Not reported
2		Card-20(22)-enolide, 3-[(6-deoxy-3,4-O-methylenehexopyranos-2-ulos-l-yl) oxy]-5,11/14-trihydroxy-12 -oxo-, (3- beta.,5-alpha., 11-alpha.)	C_30_H_40_O_11_	3.00	3.073	Not reported
3	AC (S)	Silane, [[(3alpha,5beta,20S)-pregn-11-ene-3,11,17,20-tetrayl] tetrakis(oxy)] tetrakis [trimethyl]	C_33_H_66_O_4_Si_4_	41.85	5.257	Not reported
4		2,2-Bis[4-[(4,6-dichloro-1,3,5-triazin-2-yl) oxy] phenyl]-1,1,1,3,3,3-hexafluoropropane	C_21_H_8_Cl_4_F_6_N_6_O_2_	3.00	4.241	Not reported
1	EA (D)	Phenol, 2,4-bis(1,1-dimethylethyl)	C_14_H_22_O	10.74	38.698	19
2		Curlone	C_15_H_22_O	14.44	4.233	20
3		Ar-tumerone	C_15_H_20_O	18.65	0.857	21
4		Stigmasterol	C_29_H_48_O	17.87	1.66	22
5		n-Hexadecanoic acid	C_16_H_32_O_2_	48.78	3.2	23
6		3,7,11-Tridecatrienenitrile, 4,8,12-trimethyl	C_16_H_25_N	23.94	2.567	Not reported
7		2-Methoxy-1,3-dioxolane	C_4_H_8_O_3_	39.4	0.563	Not reported
8		5H-Cyclopropa (3,4) benz(1,2-e) azulen-5-one, 1,1a-à,1b-á,4,4a,7a-à,7b,8,9,9a-decahydro-7b-à,9-á,9a-à-trihydroxy-3-hydroxymethyl-1,1,6,8-à-tetramethyl-4a-methoxy-, 9,9a-didecanoate	C_41_H_66_O_8_	58.69	2.34	Not reported
1	AC(D)	Phenol, 2-methoxy-3-(2-propenyl)	C_10_H_12_O_2_	10.73	0.678	Not reported
2		Phenol, 2,4-bis(1,1-dimethylethyl)	C_14_H_22_O	14.44	0.912	19
3		Alpha-Tocopherol	C_31_H_52_O_3_	44.65	1.612	24
4		Curlone	C_15_H_22_O	18.65	1.156	20
5		1,3-Dioxolane, 2-pentadecyl	C_21_H_40_O_4_	57.36	3.06	Not reported
6		Ar-tumerone	C_15_H_20_O	17.87	4.081	21

EA(S): Ethyl acetate sequential; AC(S): Acetone Sequential; EA(D): Ethyl acetate direct; AC(D): Acetone direct.

### Short listing of antibacterial compounds via virtual screening and pharmacokinetics

**Figure 2:**
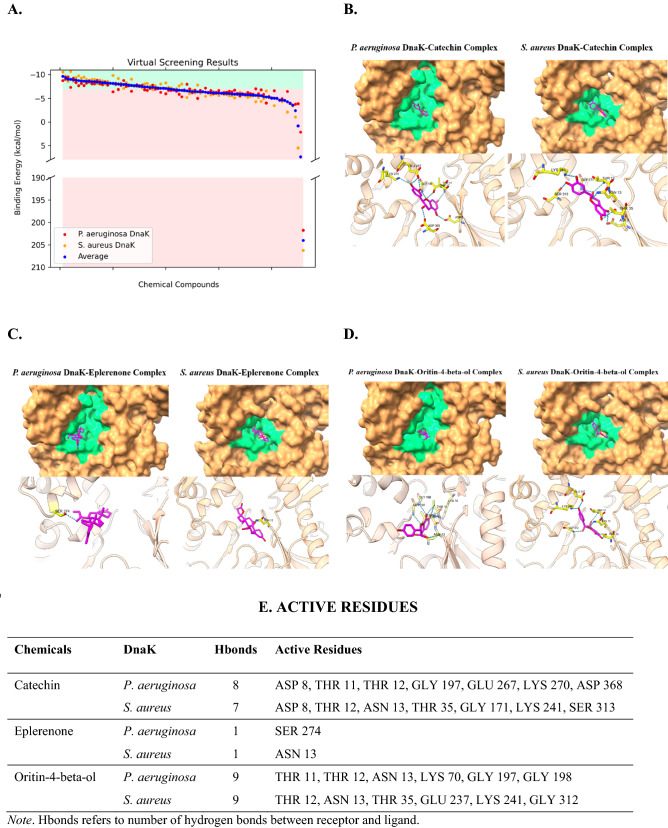
Results of virtual screening targeting *P. aeruginosa* and *S. aureus* DnaK proteins. (**A**) Distribution of binding energies plotted via Matplotlib^32^ against screened compounds. Top binders with good pharmacological properties, namely (**B**) Catechin, (**C**) Eplerenone, and (**D**) Oritin-4-beta-ol, generated using UCSF ChimeraX^33^. P2Rank predicted binding pockets were colored in green for visualization of ligands (magenta) binding to the receptor DnaK proteins (brown), with active sites (yellow) labelled. (**E**) Intermolecular hydrogen bonds tabulated with active residues listed.

We have inspected the quality of DnaK protein homology models, all of which had good steric properties (Fig. S6). Moreover, we have validated the method for virtual screening via the redocking approach, with minimal RMSD values (1.715 Å for 4B9Q and 1.427 Å for 4JNE) between reference and docked poses (Fig. S8). Upon virtual screening of the 91 chemical compounds obtained via chromatographic analyses, we considered 41 of them, with a binding energy of less than − 7 kcal/mol, as good binders (Table S5; Figs. 1, 2A). Among these, we have chosen potential drug candidates based on their predicted pharmacokinetic properties. For example, we prioritized good gastrointestinal (GI) absorption, bad BBB permeability, and non-P-glycoprotein (PGP) substrates for absorption properties. For metabolism, we prioritized non-cytochrome P450 inhibitors. Besides, we avoided violations of drug-likeness rules. To cater to the need, we considered five druggability rules, namely, the Lipinski, Ghose, Veber, Egan, and Muegge rules^25–29^. Lastly, we also prioritized higher bioavailability scores. The Abbot Bioavailability Score utilized herein was to predict chances of drug bioavailability to be more than 10% upon oral intake^30^.

Among the virtually screened compounds, we found that catechin (C), eplerenone (E) and oritin-4-beta-ol (O) stood out to be good binders with their average binding energies being − 8.205, − 7.980 and − 7.190 kcal/mol, respectively for *S. aureus* (*Sa*) and *P. aeruginosa* (*Pa*) DnaK proteins. C, E, O also exhibited good predicted pharmacological properties except that C is a PGP substrate (Fig. 1, Table S5). The binding conformations of C (Fig. 2B), E (Fig. 2C), and O (Fig. 2D) to both DnaK proteins of *Sa* (SaD) and *Pa* (PaD) showed potential structural competitive inhibition of ATP binding at the docking pocket. Moreover, we observed rich electrostatic interactions (Fig. 2E) in C and O, but not in E, having only one intermolecular hydrogen bond.

### Validation of inhibitory effects of selected compounds by Molecular Dynamics Simulation

**Figure 3:**
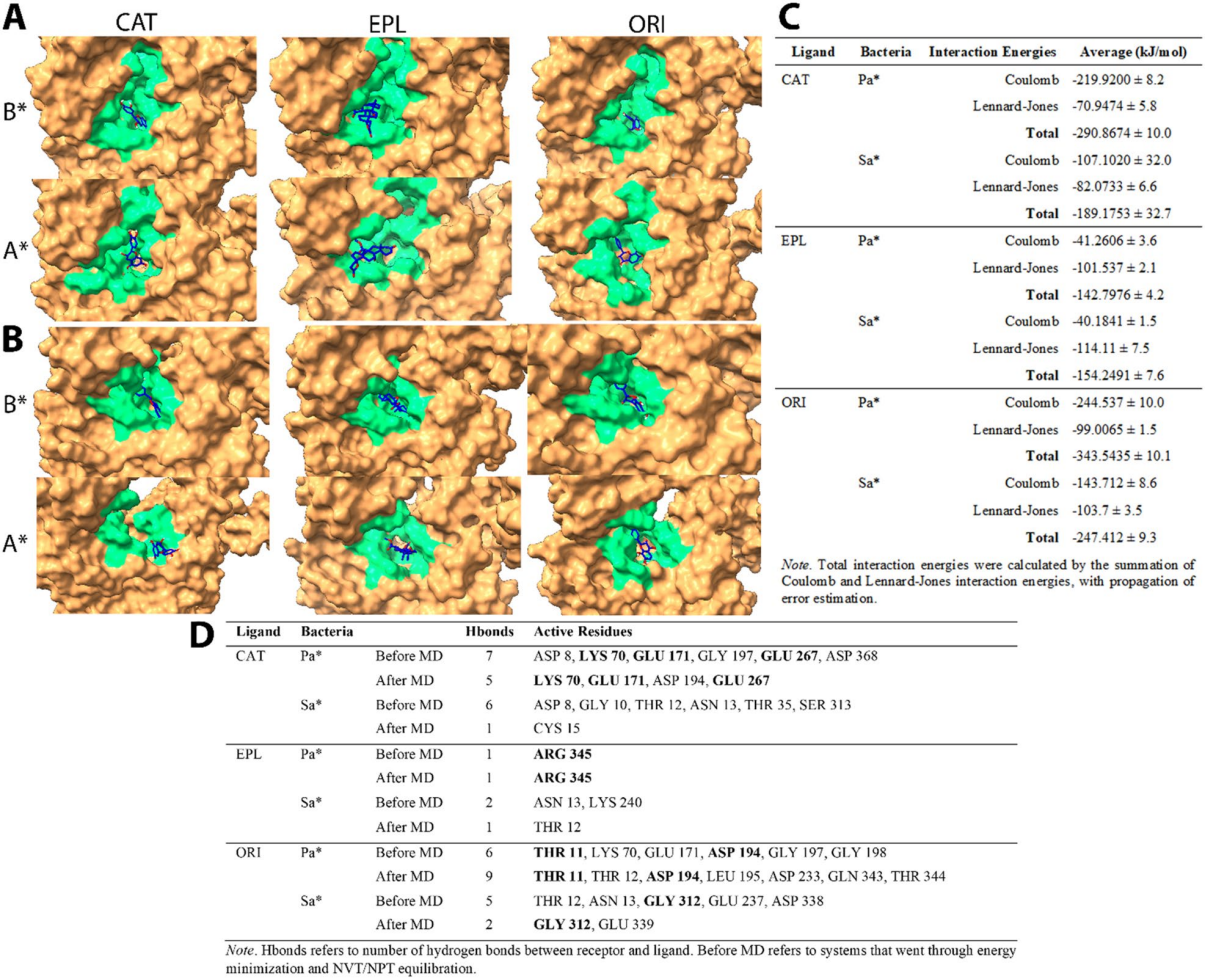
MD simulation of (**A**) *P. aeruginosa* and (**B**) *S. aureus* DnaK (brown)-Catechin(blue) complexes, of which P2Rank predicted druggable pocket residues were colored in green for better visualization. (**C**) Tabulated interaction energy values of DnaK-Catechin complexes. (**D**) Tabulated changes in number of hydrogen bonds and active residues before and after MD. *Note* B*—Before MD, A*—After MD, Pa*—*P. aeruginosa*, Sa*—*S. aureus*, CAT—Catechin, EPL—Eplerenone, ORI—Oritin-4-beta-ol. All data were generated using GROMACS^34^ in-built functions.

**Figure 4:**
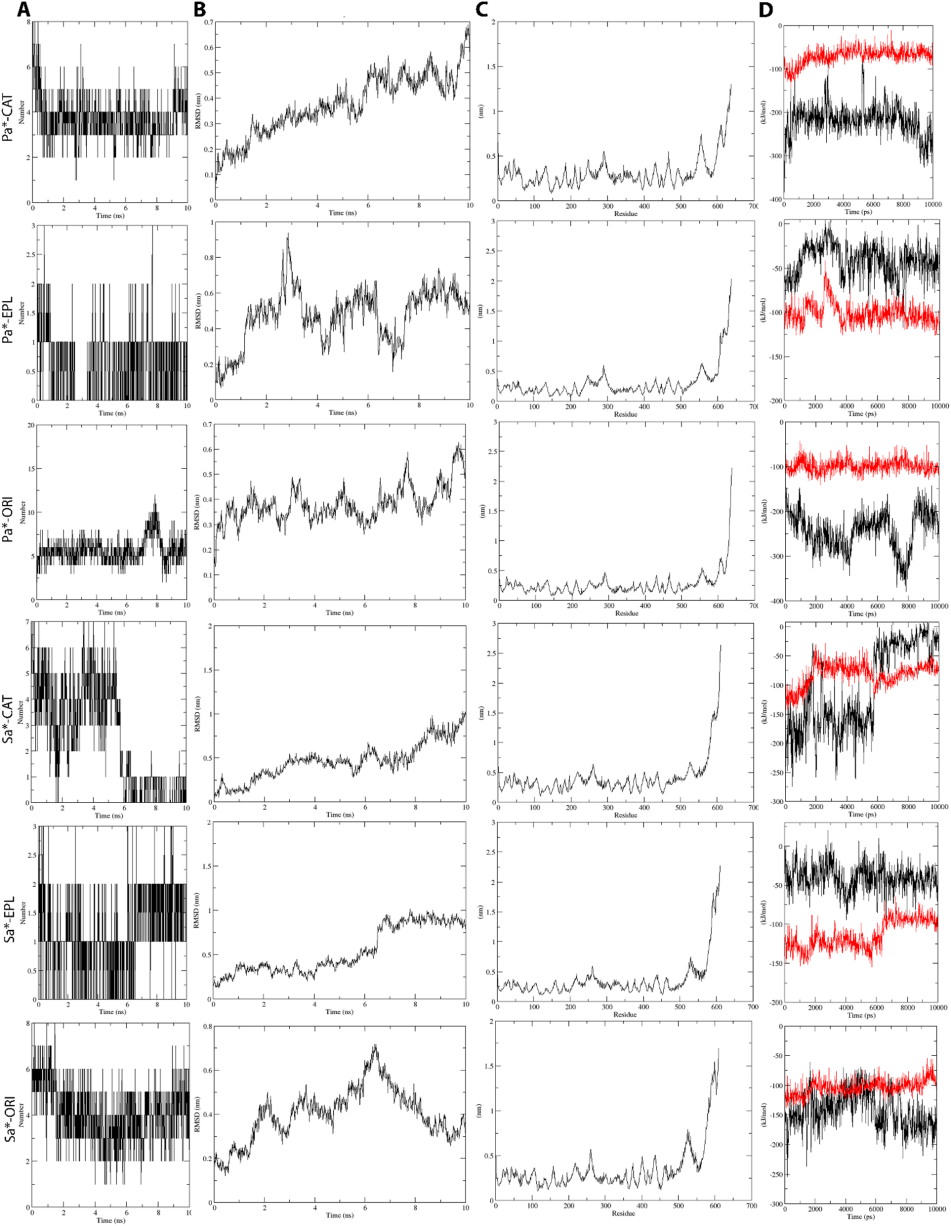
Receptor-ligand interactions over the course of MD simulation, of which (**A**) number of hydrogen bonds between receptor and ligand, (**B**) RMSD values of Catechin, (**C**) RMS fluctuation-per-residue of receptor macromolecule, and (**D**) receptor-ligand interaction energies (Black: Coulombic interaction energies, Red: Lennard–Jones energies) were computed over the course of MD. *Note* Pa*—*P. aeruginosa*, Sa*—*S. aureus*, CAT—Catechin, EPL—Eplerenone, ORI—Oritin-4-beta-ol. All data were generated using GROMACS^34^ in-built functions.

To this end, we carried out Molecular Dynamics (MD) simulations for 10 ns for C, E, and O Ligand-SaD/PaD complexes to observe ligand-receptor interactions. Throughout MD simulations, the ligands were retained in the docking pocket of respective DnaK receptors, except for C in the SaD system (CSaD) of which the ligand seemed to be escaping from the initial binding pocket (Fig. 3A, B). Moreover, the upper part of the DnaK NBD domain was completely disintegrated in CSaD. Besides, the total number of receptor-ligand intermolecular hydrogen bonds were maintained stably at around 4 and 5 in *P. aeruginosa* DnaK complexed with C (CPaD) and O (OPaD) respectively, and 4 in *S. aureus* DnaK complexed with O (OSaD) (Fig. 4A). Moreover, both the E complexes of SaD (ESaD) and PaD (EPaD) have maintained the total number of hydrogen bonds at around 1. However, in CSaD, we observed a sharp decline in the number of intermolecular hydrogen bonds at the 5 ns time point from around 4 to between 0 and 1, which can explain the escape of ligand from its initial docking pocket. We also observed stable active residues in CPaD (LYS 70, GLU 171, GLU 267), EPaD (ARG 345), and OPaD (THR 11, ASP 194) complexes, as well as in OSaD (GLY 312) complex (Fig. 3D).

In all MD simulation systems, we found that the root-mean-square fluctuations of the DnaK receptor maintained at around 0.5 nm (5 Å) except for the C-terminal end where the disordered regions were localized (Fig. 4C). Besides, we observed that the RMSD of C and E were maintained at 0.5 nm in the PaD receptor, as well as higher RMSD values of around 0.8 nm in CSaD and ESaD (Fig. 4B). The ligand RMSD were relatively lower in O, compared to others, which was 0.4 nm in both SaD and PaD cases. The interaction energies of all systems maintained stably throughout the simulation, except for the CSaD complex of which a sharp decrease of Coulomb potential can be observed at around 6 ns time point (Fig. 4D). In general, E maintained the lowest total interaction energies, followed by C and O (Fig. 3C).

## Discussion

Over the years, the commendable development in the field of virtual screening has enabled time- and cost-efficient drug discovery along with repurposing^35^. Herein, we have carried out a scaffolded approach to antimicrobial drug discovery from a yellow variety of Malaysian *N. lappaceum L.* fruit epicarp crude extracts. The first upstream set of experimental work comprised the extraction of the plant product, followed by characterization of their antimicrobial property and chromatographic identification of chemical compounds from therein. This was coupled with a downstream set of computational analyses comprising virtual screening and pharmacological predictions of extracted chemical compounds against potential drug targets. To this end, molecular dynamic simulation has taken a step forward to uncover new potent bioactive compounds which can target both gram-negative and -positive bacteria at the same time. Our study delineates a method to uncover potent chemicals which might have contributed to the antibacterial activities of plant products like *Nephelium lappaceum* epicarp, to be further utilized for drug discovery, repurposing, or other ab initio synthetic enhancements.

Extraction is the key stage to obtain the diverse bioactive chemical compounds from plant products. These chemical determinants display different solubility with different organic solvents such that screening with different solvents helps to bring forth the best one for further exploration^36^. Thus, we have utilized several organic solvents to explore the extraction of biologically active constituents. Herein, we initiated a sequential extraction process of utilizing solvents like chloroform (CF), ethyl acetate (EA), acetone (AC), ethanol (ET), methanol (MT) and water (WT), in order of their increasing polarity. Our study revealed that the yellow variety of Malaysian *N. lappaceum* epicarp crude extracts exhibited varied inhibitory activities against the six tested MDR pathogens, namely, *B. subtilis*, methicillin-resistant *S. aureus* (MRSA), *S. pyogenes, P. aeruginosa, K. pneumoniae* and *S. enterica*. Essentially, the EA(S) and AC(S) fractions notably inhibited the Gram-positive *S. pyogenes* and MRSA and the Gram-negative *P. aeruginosa* while the remaining solvent fractions responded moderately or poorly. This provided a strong clue for us to proceed for further direct extraction from EA(S) and AC(S) crude extract fractions (CEF). Thereafter, following the chromatographic analyses of HPLC, LC–MS and GC–MS of these EA(S) and AC(S) CEF, we obtained different results from the direct extract fractions of EA and AC. Interestingly, we found the CEF of EA(D) and AC(D) to be more efficient via broth dilution than the disc diffusion methods (Tables 1 and 2). This could be attributed to the following fact. The different constituents of the CEF need to diffuse slowly in agar from a liquid to solid interphase in the agar diffusion method compared to the complete liquid interphase for broth microdilution.

It is important to note that despite similar reports to our findings by Mohamed et al*.*^37^; Thitilertdecha et al*.*^38^and Tadtong et al*.*^39^, a comprehensive chemical profiling to unearth plausible determinants, potential enough against the MDR pathogens, is lacking to date. Thus, based on the prominent antibacterial effects of the EA(S) and AC(S) extracts of *N. lappaceum* fruit epicarp*,* we perceived these two fractions to harbor important bioactive molecules. Therefore, we subjected the sequential extracts of EA(S) and AC(S) fractions to HPLC analysis. The results confirmed the presence of some standard phenolic compounds with antioxidant properties namely, malic acid, vitamin C, chlorogenic acid, epigallocatechin gallate, quercetin and catechin hydrate (Table 3). Notably, we found ethyl acetate and acetone as the competent solvents to extract total flavonoid and phenolic compounds^36^. Nazir et al*.*^40,41^, however, reported the afore-mentioned compounds in various other organic solvent extracts of *Silybum marianum* and *Elaeagnus umbellate*.

To this end, an extensive spectrum of chemical classes was revealed after LC–MS analysis and included, terpenes, alkaloids, polyunsaturated and monounsaturated fatty acids among others, that were present in both the extracts. Among these, only 21 of the 54 compounds (with above 86% MFG scores) of the EA(S) fractions have been reported to possess antibacterial activities (Table S3). Interestingly, most of them have been newly reported (within the last five years) including L2^42^, L3^43^, L6^44^, L10^46^, L12^47^, L16^48^, L20^49^, L22^51^, L26^52^, L28^53^, L31^54^, L46^58^, L48^59^, L51^60^, L53^61^ and L54^59^. Others are known for some time, namely, L7^45^, L21^50^, L35^55^, L37^56^ and L45^57^^.^ Similarly, the AC(S) extract fractions contained only 10 from 44 compounds with reported antibacterial activities (Table S4). Of these, except for L24^55^ and L35^58^, all were reported recently. These included L2^44^, L5^62^, L10^48^, L13^52^, L19^54^, L36^58^, L38^59^ and L42^61^. Thus, possibilities exist for those 35 and 26 compounds from EA(S) and AC(S) fractions, respectively, with no matched identity with the library (Table S6 & S7) to be medically important, though, further characterization is required to evaluate their usages.

We have authenticated a further revelation of important biomolecules through GC–MS (Table 4) besides the HPLC and LC–MS analyses mentioned above in Tables 3 & S3–S4 respectively. Notably, we analyzed both the sequential and direct extracts of EA and AC fractions through GC–MS. Unlike the LC–MS reported compounds, however, about 50% of the chemical components, unearthed through GC–MS, are unknown for their antibacterial activity. For instance, in the case of sequential extracts, for both the EA(S) and AC(S) fractions, only 4 molecules were detected (with area % scores above 0.5%) without any a priori antibacterial activity (Table 4). Likewise, for the direct extract fractions of EA(D), only 5 out of the 8 compounds detected (with area % scores above 0.5%) were known to possess such activity. These are DGEA1^19^, DGEA2^20^, DGEA3^21^, DGEA4^22^ and DGEA5^23^. However, for the AC(D) extract fraction, 4 out of the total 6 compounds detected, were reported to possess the antibacterial effect. These are DGAC2^19^, DGAC3^24^, DGAC4^20^ and DGAC6^21^.

With a set of 91 compounds obtained through chromatographic analyses, we have conducted a computational analysis for virtual screening through molecular docking to shortlist a selective set of chemicals via pharmacokinetics consideration (Table S5). Pharmacokinetics is an important criterion when it comes to drug discovery and drug design, especially about bioavailability and toxicity. Herein, we have considered several parameters for absorption, metabolism, drug-likeness, and bioavailability for selecting the ideal drug for potential pharmacological application in the future. For instance, good GI absorption can allow absorption into the bloodstream during oral consumption, while bad blood–brain barrier (BBB) permeability can avoid interruption to the central nervous system^63^. P-glycoprotein (PGP) substrates are being actively effluxed from the cells thereby resulting in low absorption into the blood circulation^64,65^. Besides these, cytochrome P450 enzymes are crucial in the metabolism of most clinical drugs. Hence, the inhibition of cytochrome P450 enzymes can lead to decreased drug metabolism and possibly, adverse health complications, due to drug-drug interaction upon co-prescription with other drugs^66,67^. Moreover, the drug-likeness rules, such as the Lipinski rule of five, work by predicting pharmacological behavior upon oral administration based on the chemical properties of potential drugs^25^. Lastly, bioavailability takes consideration of both absorption and distribution of the drugs, of which the eventual presence in blood circulation upon oral consumption is evaluated.

DnaK protein belongs to the 70 kDa heat shock protein (HSP70) family, which functions as a molecular chaperone, mediated by its ATPase activities^68^. DnaK protein has been reported to be central in mediating bacterial stress responses. Among these, DnaK mutants have manifested an increase in antimicrobial susceptibilities and a decrease in survivability in the host^69–71^. Moreover, our previous work on whole-genome analysis (WGA) of protein interaction network (PIN) reported that DnaK protein was crucial in mediating quorum sensing in multidrug-resistant *Proteus mirabilis*^72^. Furthermore, WGA analyses of PIN from MDR pathogens like *P. aeruginosa*, S*. aureus*, *S. enterica*, *S. pneumoniae*, *P. mirabilis*, *Acinetobacter baumannii*, *Escherichia coli* and *Mycobacterium tuberculosis* revealed DnaK to be among the top 10 crucial proteins indispensable for the cellular integrity of the bacteria^73^. Also, the ATP-binding pocket of the DnaK chaperone has been indicated to be druggable and shown promise to cope with MDR in both gram negatives and positives as observed from an unpublished work of the same group of researchers. Hence, DnaK protein has been selected for the *in-silico* study, herein, as a promising drug target for MDR bacteria by inhibiting its ATP binding pocket, which can result in its impairment of chaperone function.

Through our computational screening of the chemical libraries of the *N. lappaceum L.* fruit epicarp extractions, we have shortlisted Catechin (C), Eplerenone (E), and Oritin-4-beta-ol (O) as the promising antimicrobials in combating the MDR pathogens by dint of their capacity in targeting the DnaK protein and having good pharmacological profiles. Despite being a PGP substrate, C has manifested a strong binding affinity to DnaK and therefore, can result in effective DnaK functional inhibition with a small amount. Otherwise, PGP inhibitors like C can be co-prescribed easily as it has a good metabolic profile. Moreover, C has been well-characterized for its antibacterial activities and known for its ability to cause leakage of bacterial cellular contents along with increased intracellular reactive oxygen species production in both gram negatives and positives^74,75^. However, the biological targets of C have not been described. As DnaK protein is crucial in bacterial stress response, by inhibiting the DnaK chaperone function, the bacterial cellular and biomolecular integrity can be effected upon receiving environmental oxidative stress. Herein, we showed that in *P. aeruginosa*, C could bind stably to the ATP-binding pocket of DnaK throughout the MD simulation with 3 stable active residues (LYS 70, GLU 171, and GLU 267), while maintaining the ATP-bound conformation of the DnaK protein without the necessity for ATP binding (Figs. 2B, 3A). This reflected the inability of the ATP molecules to bind the CPaD (Catechin-bound DnaK protein of *P. aeruginosa*) as also a complete halting of the normal DnaK chaperone function via conformational changes ensuing ATP hydrolysis. However, C could not inhibit SaD (DnaK of *S*. *aureus*) the same way, due to its inability to maintain the integrity of the NBD domain and thereby escaping from the binding pocket. It is this binding pocket that allows subsequent binding of ATP molecules on DnaK to continue the chaperone function. On the contrary, herein, we present the discovery of two novel potential compounds, E and O, whose antibacterial activities have not been reported and/or described earlier. Notably, E has been widely utilized in cardiovascular implications and as diuretics^52,76^. O, however, has not been explored to confer any biological significance. Despite that, it is notable that the chemical structure of O is analogous to C (Fig. 1), with the sites of hydroxylation being slightly different.

Throughout the molecular dynamics simulation (MDS) processes, we can only observe 1 or 2 hydrogen bonds in EPaD and ESaD, which suggested weak protein–ligand electrostatic interactions. This can be explained by the chemical structure of E, being crowded with carbonyls and ethers which are weak bases, and hydroxyl groups are lacking. The ligand, however, has been retained in the docking pocket throughout MDS. This probably suggests that hydrophobic (van der Waals) interactions were dominant in this case. This was reflected through the intermolecular interaction energies (Fig. 4D), of which the Lennard–Jones potentials were much higher than Coulomb potentials in Eplerenone-DnaK (ED) complexes, while the reverse was observed in for C and O. Moreover, the binding conformation of E in PaD did not “cover-up” completely at the binding site of phosphate groups of the ATP for which further wet-lab confirmation is required. Furthermore, among the three ligands simulated, O manifested the best binding capabilities to both PaD and SaD with rich intermolecular electrostatic interactions and the highest total interaction energies. After MDS, the active residues THR 11 and ASP 194 were retained in OPaD, while GLY 312 was retained in OSaD. Again, despite being structurally analogous to C, O manifested good predicted pharmacological properties in all the aspects considered. Therefore, with better binding capabilities to DnaK receptor and pharmacological properties, herein we report O to be a more potent antibacterial compound compared to the well-known C, which is active against both the gram-positive and -negative bacteria.

In the end, E, the compound not reported earlier to exhibit antibacterial properties against the tested promising pathogens MRSA and *P. aeruginosa*, demands a separate focus. Importantly, E has been found in both the EA(S) and AC(S) CEF. However, the EA(S) showed no activity compared to the AC(S) CEF. This might probably be attributed to the interference of other chemicals in that EA(S) which might not have been the case for AC(S), probably, facing no interference and thus, showing activities. Thus, E can be a probable candidate as projected through our *in-silico* studies comprising screening of pharmacological properties followed by molecular dynamics simulation.

## Conclusion

Our findings reinstate the promising antibacterial effects, of the yellow variety of Malaysian Rambutan *(N. lappaceum L.)* fruit epicarp crude extracts, against selected Gram-positive and Gram-negative MDR pathogens. In this context, particularly ethyl acetate and acetone (sequential and direct) extracts demonstrated remarkable antibacterial effects toward at least MRSA and *P. aeruginosa* among the six tested MDR pathogens, while remaining fractions including, chloroform, ethanol, methanol and water did not exhibit such potential. Nevertheless, we present the epicarp of *N. lappaceum* as a novel source for antibacterial compounds projecting catechin, eplerenone and oritin-4-beta-ol with high potential for the development of pharmaceutically valuable future drugs. Further studies are mandatory to separate the specifically mentioned three compound(s) responsible for the desired effects and to develop our knowledge on the other unseen potentials in *N. lappaceum.*

## Materials and methods

### Solvents

For the preparation of crude extracts, we have used all solvents, of HPLC grades. In the order of increasing polarities, these were Chloroform (99.9%, Sigma-Aldrich, LiChrosolv, Malaysia), Ethyl acetate, Acetone (99.5% Chemiz, Malaysia), Ethanol, Methanol (99.8%, ChemAR, Systerm, Malaysia) and double distilled Milli-Q Type 1 water (MilliporeMerck, Germany). For LC–MS and GC–MS studies, we have used the solvents of MS grades.

### Plant product

We purchased the yellow variety fruits of *N. lappaceum L.* from the local marketplace, Bandar Sunway, Selangor, Malaysia. We prepared the Herbarium voucher and deposited at Sunway University, Selangor Darul Ehsan, Malaysia. Thereafter, for our research, we carefully observed the fruit characters of rambutan and selected the epicarp according to the Descriptor for Rambutan^77^.

Tested microorganisms.For our study, we obtained six clinical isolates from the Department of Biological Sciences, Sunway University, Malaysia. These were *Streptococcus pyogenes* (ATCC-49399), *Bacillus subtilis* (ATCC-11774)*,* methicillin-resistant *Staphylococcus aureus* (MRSA) (MTCC-381123), *Pseudomonas aeruginosa* (ATCC-10145), *Klebsiella pneumoniae* (ATCC-700603) and *Salmonella enterica* (ATCC-14028). We tested all these six bacterial strains to be resistant to at least five of the ten antibiotics tested for their resistivity/sensitivity profile and thus, considered them to be multidrug-resistant (Table S8).

### Preparation of crude extracts

We prepared the epicarp crude extracts following the method of Do et al*.*^78^ using the solvents mentioned earlier for the direct extracts. For the sequential method of extraction, we used the mentioned solvents in the order of their increasing polarity viz*.* chloroform < ethyl acetate < acetone < ethanol < methanol < water. In both cases, essentially, we removed the peels of *N. lappaceum* from the fruit and washed thoroughly with running, followed by, distilled water to remove contaminants and thereafter dried using a freeze-dryer. We ground the dried peels into a fine powder using an electric grinder. To produce different fractions of crude extracts, we extracted 10 g of powder in 100 ml of selected solvents. Thereafter, we mixed the solution thoroughly by using an incubator shaker (Yihder LM-530D Incubator Shaker, Taiwan) for 24 h. To separate supernatant, we centrifuged the solution (Eppendorf 5810 R Centrifuge, Germany) at 4000 rpm for 10 min at 4 °C to eliminate the leftover fine sediments. Finally, we concentrated the solvent extracts using a Rotary evaporator, and further with a vacuum concentrator until a viscous extract was obtained. We stored all the extracts at 4 °C for future experiments.

### Potential *in-vitro* antibacterial activities of yellow rambutan fruit epicarp extracts

#### Disc diffusion assay

We consistently swabbed the seed culture of the tested pathogen on an agar plate. Then, we separately impregnated sterilized blank paper discs with different crude extract fractions and placed them on the agar plate. We incubated the plates at 37ºC for 16 h. We noted the antibacterial activity by measuring the diameter of the inhibition zone. We used gentamicin (10 µg/disc) as positive control and kept DMSO (< 1%) as a negative control. We ensured that all the experiments had technical triplicates and we performed them twice to render two biological replicates.

#### Broth dilution assay

We used a broth micro-dilution method to evaluate the minimum inhibitory concentration (MIC) values of crude extracts using Clinical & Laboratory Standards Institute (CLSI) procedures. Essentially, we added each extract (5 μl) into the wells of a 96 well plate comprising 10^5^ CFU/ml bacterial cells. We incubated the 96 well plates at 37 °C for 16 h. We kept the final concentrations ranging from 250 to 2000 µg/ml. In each test, we included three controls comprising, gentamicin 10 µg/ml (as positive), DMSO < 1% final concentration (as solvent) and bacterial inoculum (as negative). We have taken the MIC value as the lowest concentration of the tested extract showing inhibitory effect against the pathogens, recorded via the Microplate reader (TECAN, Infinite-M200-PRO). We confirmed all tests, having technical triplicates, twice. We observed promising results for both the fractions of ethyl acetate and acetone extracts with which we carried out all chromatographic analyses.

#### Statistical tests

In the present study, we performed all the tests in triplicates and expressed the data obtained as the mean ± standard deviation (S.D). We determined the P values using the student’s T-test, two-tailed distribution, (*) is *P* ≤ 0.05. These have been reflected in Tables 1 and 2 and Figure S2.

### Exploration of chemical constituents through chromatographic analyses

#### High-performance Liquid Chromatography (HPLC)

We used the ethyl acetate and acetone extracts as samples for qualitative phytochemical screening via HPLC via the Agilent-1260 infinity system, according to the reported method of Zeb^79^. Briefly, we mixed one-gram sample extract in methanol and water (1:1; 20 mL; v/v) and heated at 70˚C for 1 h in a water bath. We centrifuged this mixture at 4000 rpm for 10 min and filtered 2 ml of the supernatant into HPLC vials through Whatman filter paper. We performed the separation via the Agilent-Zorbax-Eclipse column (XDB-C18). Column gradients system comprised solvent B and C. Solvent B consisted of deionized water: methanol: acetic acid having a ratio of 180: 100: 20; v/v while solvent C had deionized water: methanol: acetic acid in the ratio of 80: 900: 20; v/v. We started the gradient system by solvent B for 100%, 85%, 50% and 30% at 0, 5, 20 and 25 min followed by solvent C (100%) from 30 to 40 min. Elution occurred after 25 min. We set the ultraviolet array detector (UVAD) at 280 nm for the antioxidants analysis and documented the chromatogram using retention times. We carried out the UV spectra of compounds and accessible standards along with quantification by taking the per cent peak area. We measured the quantity of the antioxidants by the formula:

1 $$Cx = \frac{{Ax \times Cs\left({\frac{{{\upmu }{\text{g}}}}{{{\mathrm{ml}}}}} \right) \times V\left( {{\text{ml}}} \right)}}{{As \times Sample~\left( {{\text{wt}}{{.}}~\;{\text{in}}\;~{\text{g}}} \right)}}$$

*Cx* = Sample concentration; *As* = Standard peak area; *Ax* = Sample peak area; *Cs* = Standard concentration (0.09 µg/ml).

#### Liquid chromatography and mass spectrometry (LC–MS)

We analyzed a mixture of standards and new metabolites found in the ethyl acetate and acetone fractions via LC–MS, exactly as per the method reported by Yap et al*.*^80^. To eradicate systematic errors, we used a reference solution with the two ions, with m/z of 121.0508 and 92,266.0097, being selected for mass calibration. Finally, we ran the mass spectra for the compounds present in ethyl acetate (EA) and acetone (AC) fractions against the database of NIST (National Institutes of Standard and Technology, Gaithersburg, MD, USA) Mass Spectral Search Program-2009 version 2 for the documentation of homologous compounds over Agilent Mass-Hunter Qualitative Analysis B.05.00 software.

#### Gas chromatography–mass spectrometry (GC–MS)

We subjected the ethyl acetate and acetone fractions to gas chromatography-mass spectrometry (GC–MS) analysis, using Agilent technologies model 7890B GC System coupled with Pegasus HT High Throughput TOFMS (Leco Corp., MI, USA). We injected an aliquot of an extract of 1 ml into the GC–MS apparatus. Next, we used Agilent J&W HP-5MS (phenylmethyl siloxane, length 30 m, Dia. 0.32 mm, Film, 0.25 µm) analytic column to separate components under an inert atmosphere of helium (1.5 mL/min). Other standardized parameters utilized during the process: oven temperature of 80 °C (2 min) was increased to a temperature of 300 °C at the rate of 3 °C/min, solvent delay time was 5 min, inlet line temperature was 225 °C, and ion source temperature was 250 °C. Mass spectra were taken at 70 eV and the acquisition mode-scan was 20–1000 amu while sixty-four (64) minutes was the GC run time. We achieved the interpretation of mass spectrum and the documentation of phytochemicals present in the fractions via the database of NIST libraries.

### Virtual screening of chemical determinants from chromatographic analyses

#### In silico protein model generation

We have chosen *S. aureus* (*Sa*) and *P. aeruginosa* (*Pa*) as gram-positive and gram-negative bacterial representatives for computational analyses of DnaK protein binding. 3D structures of DnaK proteins, from the aforesaid species, were generated via homology modelling using MODELLER version 9.24^81^. DnaK has two conformations, namely, the open or ATP-bound and the closed or ADP-bound conformation^65^. Herein, we focused on the open conformation of DnaK, to identify potential competitive inhibitors of ATP to prevent the proper functioning of DnaK protein.

We have obtained the protein sequences of *Sa* and *Pa* DnaK from UniProtKB with accession IDs of Q2FXZ2 and A6VCL8^82^, respectively. To search for suitable homology modelling templates, we utilized both NCBI BLASTp and the MODELLER in-built build_profile.py^81,83^. For *Pa* DnaK (PaD), the templates were full-length ATP-bound *E. coli* (*Ec*) DnaK protein structures (PDB ID: 5NRO, Chain: A, Query Coverage (QC): 94%, Percent Identity (PI): 79.50%, Resolution (R): 3.25 Å; PDB ID: 4JNE, Chain: A, QC: 94%, PI: 78.80%, R: 1.96 Å; and PDB ID: 4B9Q, Chain: A, QC: 94%, PI: 77.96%, R: 2.40 Å). For *Sa* DnaK (SaD), besides the afore-mentioned *Ec* DnaK (EcD) models, we selected one additional template, from *Geobacillus kaustophilus* DnaK protein (PDB ID: 2V7Y, Chain: A), due to the high percentage of sequence identity expected as per the gram-positive character of *S. aureus* and *G. kaustophilus*. As this template structure was in the closed conformation and we were only interested in the open conformation, only the Nucleotide Binding Domain (NBD, residues 1 to 350 in template model) which does not differ much in both conformations, were taken into consideration for homology modelling, and the remaining C-terminal residues modelling were guided by the *Ec* models to shape an open conformation. Therefore, the templates for SaD were (PDB ID: 2V7Y, Chain: A, Template Residues: 1–350, QC: 57%, PI: 83.19%, R: 2.37 Å; PDB ID: 5NRO, Chain: A, QC: 93%, PI: 56.19%, R: 3.25 Å; PDB ID: 4JNE, Chain: A, QC: 92%, PI: 55.54%, R: 1.96 Å; and PDB ID: 4B9Q, Chain: A, QC: 94%, PI: 55.43%, R: 2.40 Å). The template sequences were aligned with the target sequence for homology modelling via the built-in function of MODELLER (Fig. S6A). 5 homology models were generated for each protein of SaD and PaD, and the models with the lowest DOPE (discrete optimized protein energy) scores were selected for downstream virtual screening for both. We then validated the SaD and PaD homology models via Swiss-Model Structure Assessment and SAVES v5.0 servers^84^ (Fig. S6).

#### Druggable pocket validation

To validate the druggability of the ATP docking pocket, we have conducted ligand binding site prediction using P2Rank from PrankWeb server^85^. P2Rank predicts the chemical druggability on protein solvent-accessible surface via a non-templated machine learning approach. The ATP binding pocket was predicted to be druggable and ranked first in both cases of SaD and PaD (Table S9; Fig. S7). Thus, we further considered these pockets from the SaD and PaD complexes to be targeted for virtual screening.

#### Molecular docking with chemical determinants

We utilized the POAP pipeline^86^ for an in silico virtual screening of the chemical compounds obtained through different chromatographic separation. We have obtained the SMILES notations of these compounds, and generated their 3D models (in mol2 format) through the POAP Ligand Preparation pipeline. To this end, we utilized Chimera to generate physiological protonation states of the ligands, and PDBQT files were prepared^87^. We also carried out ligand optimizations via the POAP Ligand Preparation pipeline utilizing the MMFF94 force field which is being optimized for drug-like organic molecules and molecular docking^88^. Out of the 50 conformers, generated for each ligand through the Weighted Rotor Search approach, only the best conformers were retained. Finally, we have subjected the ligands to energy minimization for 5000 steps by the conjugate algorithm.

We have prepared the macromolecule receptors, of the SaD and PaD proteins, using AutoDockTools. We utilized AutoDock 4.2, aided by the POAP pipeline, for the virtual screening process^89^. For AutoDock parameters, we have set 100 generations of Lamarckian Genetic Algorithm for each protein–ligand complex. To fit in the previously predicted pocket, we adjusted the docking grids into squares of 24 Å with x, y, z coordinates of 17.647, 75.43, 27.766, and 18.069, 74.299, 28.532, for SaD and PaD, respectively. For the silicon-containing compound among the set of ligands, we separately carried out molecular docking with AD4.1_bound parameter file, obtained from AutoDock, wherein we added the parameters for silicon atoms (Rii = 4.3; eii = 0.402)^90^.

We have validated the docking methodology via redocking of experimentally confirmed and deposited structures with the reference ligand (Fig. S8). Therein, we have retrieved the ATP-bound *E. coli* DnaK crystallized structures from PDB (PDB ID: 4B9Q, CHAIN ID: A; PDB ID: 4JNE, CHAIN ID: A). The molecular docking search grids were squares of 24 Å, in x, y, z coordinates of 108.958, 73.922, 100.622, and 19.081, 76.887, 31.16, for 4B9Q and 4JNE respectively.

#### Pharmacological properties screening

Using SwissADME^91^, we have carried out predictions on the pharmacological properties, encompassing pharmacokinetics, drug-likeness, and molecular information, for each chemical compound.

#### Molecular dynamics simulation

Ensuing virtual and pharmacological screenings, we rationally selected potential drug candidates to undergo molecular dynamics (MD) simulation via GROMACS version 2019.3^34^. We have utilized the CHARMM36 force field of version July 2020, along with the TIP3P water model, for macromolecule processing^92^. We used Avogadro software for mol2 format conversion and complete protonation (protonation of non-polar atoms)^93^. We also used a Perl script, sort_mol2_bonds.pl, written by Justin Lemkul for bond order arrangements in ligand mol2 files. Then, we generated the topologies of the ligand models through the CGenFF server, and utilized a python script (cgenff_charmm2gmx.py) to convert topologies for CHARMM to GROMACS^94^. We carried out solvation in a dodecahedron box ranged 1.0 Å from the protein–ligand complex. The system was then ionized to achieve electrostatic neutralization. Subsequently, we subjected the system to energy minimization via the steepest descent algorithm until convergence at a maximum force of less than 1000 kJ mol^−1^ nm^−1^ (Fig. S9). Herein, we have monitored the potential energy shifts of the systems.

We carried out equilibration of the systems via NVT and NPT ensembles for 50,000 steps (100 ps), with temperature, pressure, and density shifts being monitored therein. Subsequently, we have carried out the production MD simulations for 5,000,000 steps (10 ns) to observe protein–ligand interactions. We computed the RMSD (Root Mean Square Deviation) values of ligands and receptors, number of hydrogen bonds between ligands and receptors, and ligand-receptor interaction energies (Coulombic interaction energies and Lennard–Jones energies) throughout the MD simulations. We have also computed thetotal interaction energies, and estimated the errorsvia error propagation by addition.

#### Generation of graphical illustrations

We utilized Matplotlib, a python library, to tabulate binding energies of all screened compounds^31^. We generated all 3D structural images using UCSF ChimeraX^32^, and 2D chemical structures using MarvinSketch^33^. Finally, we retrieved the figures for MD simulation analyses from the GROMACS in-built functions^34^.

## Supplementary Information

Supplementary Information.
