# Supplementary material for: A scaffolded approach to unearth potential antibacterial components from epicarp of Malaysian Nephelium lappaceum L
Source: Sci Rep. 2021 Jul 5;11:13859. doi: 10.1038/s41598-021-92622-0 (PMC8257635; doi:10.1038/s41598-021-92622-0)
Supplement: Supplementary file 1 — Supplementary Information. [file 41598_2021_92622_MOESM1_ESM.docx]

**A Scaffolded Approach to Unearth Potential Antibacterial Components from Epicarp Of Malaysian Nephelium Lappaceum L.**

**Authors:** Ali Asghar^1^, Yong Chiang Tan^1^, Mohammad Zahoor^2^, Syafiq Asnawi^3^, Yoon-Yen Yow^1^, Ezzat Khan^4^ and Chandrajit Lahiri^1*^

**Addresses:**

^1^ Department of Biological Sciences, Sunway University, Petaling Jaya, Malaysia

^2^ Department of Biochemistry, University of Malakand, Chakdara, Pakistan

^3^ Jeffrey Cheah School of Medicine and Health Science, Monash University Malaysia, Petaling Jaya, Malaysia

^4^ Department of Chemistry, University of Bahrain, Sakhir, Bahrain.

**Correspondence:** *Chandrajit Lahiri, **E-mail:** [chandrajitl@sunway.edu.my](mailto:chandrajitl@sunway.edu.my)

**Phone:** +60 3-7491 8622**Fax:** +6 03 5638 7177

**Co-Authors:** Ali Asghar, E-mail: [ali.a13@imail.sunway.edu.my](mailto:ali.a13@imail.sunway.edu.my)

Yong Chiang Tan, E-mail: [alexyc991@gmail.com](mailto:alexyc991@gmail.com)

Mohammad Zahoor, E-mail: [mohammadzahoorus@yahoo.com](mailto:mohammadzahoorus@yahoo.com)

Syafiq Asnawi, E-mail: [syafiq.asnawi@monash.edu](mailto:syafiq.asnawi@monash.edu)

Yoon-Yen Yow, E-mail: [yoonyeny@sunway.edu.my](mailto:yoonyeny@sunway.edu.my)

Ezzat Khan, E-mail: [ezkhan@uob.edu.bh](mailto:ezkhan@uob.edu.bh)

**SUPPLEMENTARY MATERIALS:**

**Table S1.** Antibacterial activity of *N. lappaceum* sequential extracts based on disc diffusion method

| **Zones of Inhibition (mm)** | | | | | | |
| --- | --- | --- | --- | --- | --- | --- |
| **Microorganisms** | **CF** | **EA** | **AC** | **ET** | **MT** | **WT** |
| *B. subtilis* | - | - | - | - | - | - |
| MRSA | - | - | - | - | - | - |
| *S. pyogenes* | - | - | - | - | - | - |
| *P. aeruginosa* | - | - | - | - | - | - |
| *K. pneumonia* | - | - | - | - | - | - |
| *S. enterica* | - | - | - | - | - | - |

‘‘**-**’’, no inhibition zone, CF; Chloroform, EA; ethyl acetate, AC; acetone, ET; ethanol, MT; methanol, WT; water extracts

**Table S2**. Percentage of antibacterial activity of sequential extracts by broth dilution method

| **% Inhibition of all tested pathogen** | | | | | | |
| --- | --- | --- | --- | --- | --- | --- |
| **Extracts** | ***MRSA*** | ***B. subtilis*** | ***S. pyogenes*** | ***P. aeruginosa*** | ***S. enterica*** | ***K. pneumonia*** |
| **CF** | 40 | 05 | 35 | 30 | 10 | 05 |
| **ET** | 30 | 35 | 35 | 30 | 10 | 20 |
| **MT** | 20 | 40 | 45 | 40 | 10 | 20 |
| **WT** | 10 | 05 | 15 | 20 | 05 | 10 |

CF; Chloroform, EA; ethyl acetate, AC; acetone, ET; ethanol, MT; methanol, WT; water

**Exploration of other chemical determinants through Liquid Chromatography–Mass Spectrometry (LC-MS) Analysis**

**Table S3**. Identified compounds existing in *N. lappaceum* ethyl acetate extract identified by LC-MS analysis

| **No** | **m/z** | **Identified compounds** | **Molecular Formula** | **RT (Min.)** | **Mass** | **Score (DB)** | **Antibacterial activity**  **reports** |
| --- | --- | --- | --- | --- | --- | --- | --- |
| 1 | 104.1066 | 2-Amino-3-methyl-1-butanol | C_5_ H_13_ N O | 1.517 | 103.0992 | 95.45 | Not reported |
| 2 | 122.0806 | Tromethamine | C_4_ H_11_ N O_3_ | 1.5 | 121.0731 | 90.86 | 38 |
| 3 | 139.0387 | p-Salicylic acid | C_7_ H_6_ O_3_ | 9.772 | 138.0315 | 86.19 | 39 |
| 4 | 157.0494 | Iretol | C_7_ H_8_ O_4_ | 9.771 | 156.0422 | 86.71 | Not reported |
| 5 | 175.0599 | 6,7-dihydroxy-4-oxo-2-heptenoic acid | C_7_ H_10_ O_5_ | 15.388 | 174.0527 | 99.23 | Not reported |
| 6 | 195.0867 | Quebrachitol | C_7_ H_14_ O_6_ | 1.604 | 194.0794 | 99.12 | 40 |
| 7 | 217.0681 | D-Glucoside | C_7_ H_14_ O_6_ | 1.588 | 194.079 | 86.27 | 41 |
| 8 | 219.0267 | L-Galactose | C_6_ H_12_ O_6_ | 1.546 | 180.0641 | 87.34 | Not reported |
| 9 | 226.1289 | Ethyl beta-D-glucopyranoside | C_8_ H_16_ O_6_ | 2.196 | 208.0948 | 96.07 | Not reported |
| 10 | 233.0436 | Caffeine | C_8_ H_10_ N_4_ O_2_ | 1.593 | 194.0808 | 88.04 | 42 |
| 11 | 247.0934 | 5,6-Dihydrouridine | C_9_ H_14_ N_2_ O_6_ | 1.737 | 246.086 | 95.81 | Not reported |
| 12 | 253.1284 | 2,3-Butanediol glucoside | C_10_ H_20_ O_7_ | 2.548 | 252.121 | 96.45 | 43 |
| 13 | 279.1554 | Mycosporine | C_11_ H_19_ N O_6_ | 3.449 | 261.1216 | 99.21 | Not reported |
| 14 | 284.1347 | 1-(3-Carboxypropyl)-3,7-dimethylxanthine | C_11_ H_14_ N_4_ O_4_ | 2.801 | 266.101 | 96.71 | Not reported |
| 15 | 291.0862 | Oritin-4beta-ol | C_15_ H_14_ O_6_ | 10.143 | 290.0788 | 96.06 | Not reported |
| 16 | 303.0151 | Ellagic acid | C_14_ H_6_ O_8_ | 11.033 | 302.0077 | 92.82 | 44 |
| 17 | 303.0425 | 2'-Hydroxyfurano [2'',3'':4',3’] chalcone | C_17_ H_12_ O_3_ | 11.37 | 264.0795 | 87.51 | Not reported |
| 18 | 315.0714 | Salicyl phenolic glucuronide | C_13_ H_14_ O_9_ | 9.404 | 314.0645 | 94.36 | Not reported |
| 19 | 316.2846 | (4OH,8Z, t18:1) sphingosine | C_18_ H_37_ N O_3_ | 14.897 | 315.2773 | 98.48 | Not reported |
| 20 | 318.3004 | Phytosphingosine | C_18_ H_39_ N O_3_ | 15.37 | 317.2931 | 99.41 | 45 |
| 21 | 321.0246 | Niflumic Acid | C_13_ H_9_ F_3_ N_2_ O_2_ | 9.864 | 282.0617 | 90.14 | 46 |
| 22 | 339.1233 | (-)-Glyceollin I | C_20_ H_18_ O_5_ | 16.008 | 338.1161 | 96.26 | 47 |
| 23 | 365.1559 | Kinetin Riboside | C_15_ H_17_ N_5_ O_5_ | 8.704 | 347.1223 | 95.94 | Not reported |
| 24 | 369.1334 | Aurmillone | C_21_ H_20_ O_6_ | 16.218 | 368.1263 | 98.52 | Not reported |
| 25 | 386.1596 | Papaverrubine B | C_21_ H_23_ N O_6_ | 9.795 | 385.1525 | 99.08 | Not reported |
| 26 | 415.212 | Eplerenone | C_24_ H_30_ O_6_ | 16.63 | 414.2048 | 98.7 | 48 |
| 27 | 420.1864 | Benzyl O-[arabinofuranosyl-(1->6)-glucoside] | C_18_ H_26_ O_10_ | 9.77 | 402.1527 | 97.76 | Not reported |
| 28 | 435.0926 | Avicularin | C_20_ H_18_ O_1_  _1_ | 11.242 | 434.0856 | 96.85 | 49 |
| 29 | 437.3414 | Demethyl phylloquinone | C_30_ H_44_ O_2_ | 16.163 | 436.3343 | 92.02 | Not reported |
| 30 | 449.1082 | 6-Hydroxyluteolin 5-rhamnoside | C_21_ H_20_ O_11_ | 11.369 | 448.101 | 98.51 | Not reported |
| 31 | 455.3518 | Bryononic acid | C_30_ H_46_ O_3_ | 14.871 | 454.3448 | 99.16 | 50 |
| 32 | 465.1033 | 8-Hydroxyluteolin 8-glucoside | C_21_ H_20_ O_12_ | 10.908 | 464.0959 | 99.07 | Not reported |
| 33 | 565.3885 | 3beta-Acetoxy-11alpha-methoxy-12-ursen-28-oic acid | C_34_ H_54_ O_5_ | 15.436 | 542.3995 | 87.71 | Not reported |
| 34 | 579.1496 | Apigenin 7-(3''-p-coumaroylglucoside) | C_30_ H_26_ O_12_ | 9.851 | 578.1415 | 88.85 | Not reported |
| 35 | 593.2761 | Pheophorbide a | C_35_ H_36_ N_4_ O_5_ | 22.878 | 592.2689 | 99.21 | 51 |
| 36 | 611.1398 | Isoorientin 6''-O-caffeate | C_30_ H_26_ O_14_ | 12.092 | 610.1328 | 97.43 | Not reported |
| 37 | 611.1602 | Robinetin 3-rutinoside | C_27_ H_30_ O_16_ | 10.634 | 610.1528 | 98.45 | 52 |
| 38 | 619.4206 | 19-Methoxypomolic acid 3-arabinoside | C_36_ H_58_ O_8_ | 15.458 | 618.4135 | 98.04 | Not reported |
| 39 | 629.4043 | Zizyphine A | C_33_ H_49_ N_5_ O_6_ | 15.351 | 611.3704 | 89.69 | Not reported |
| 40 | 652.1142 | Punicacortein A | C_27_ H_22_ O_18_ | 9.86 | 634.0802 | 99.1 | Not reported |
| 41 | 700.2812 | Bruceoside A | C_32_ H_42_ O_16_ | 10.401 | 682.2469 | 95.47 | Not reported |
| 42 | 751.462 | Oleanolic acid 3-[glucosyl-(1->4)-xyloside] | C_41_ H_66_ O_12_ | 14.874 | 750.4547 | 99.01 | Not reported |
| 43 | 757.4516 | beta-Hederin | C_41_ H_66_ O_11_ | 15.346 | 734.4612 | 87.4 | Not reported |
| 44 | 775.4626 | Hebevinoside VI | C_41_ H_68_ O_12_ | 15.387 | 752.4731 | 94.26 | Not reported |
| 45 | 783.0668 | Emblicanin A | C_34_ H_22_ O_22_ | 9.678 | 782.0598 | 97.26 | 53 |
| 46 | 785.0833 | Pedunculagin | C_34_ H_24_ O_22_ | 10.438 | 784.0757 | 98.63 | 54 |
| 47 | 793.4741 | Sanguisorbin E | C_43_ H_68_ O_13_ | 15.443 | 792.4665 | 99 | Not reported |
| 48 | 804.1241 | Tercatain | C_34_ H_26_ O_22_ | 10.393 | 786.0905 | 97.33 | 55 |
| 49 | 823.0995 | 1,3,4,5-Tetra-O-galloylquinic acid | C_35_ H_28_ O_22_ | 10.781 | 800.1101 | 90.85 | Not reported |
| 50 | 826.1326 | Reduced flavine adenine dinucleotide (FADH2) | C_27_ H_35_ N_9_ O_15_ P_2_ | 9.346 | 787.1699 | 83.43 | Not reported |
| 51 | 883.5037 | Pitheduloside B | C_46_ H_74_ O_16_ | 14.87 | 882.4964 | 97.93 | 56 |
| 52 | 907.5043 | 3-O-(Rhaa1-4(Rhaa1-2) Glcb) -(25R)-spirost-5-en-3beta-ol | C_46_ H_76_ O_16_ | 15.368 | 884.515 | 96.24 | Not reported |
| 53 | 970.1146 | Geraniin | C_41_ H_28_ O_27_ | 9.676 | 952.0808 | 98.43 | 57 |
| 54 | 972.1301 | Chebulagic acid | C_41_ H_30_ O_27_ | 10.431 | 954.0955 | 94.85 | 55 |

**Table S4.** Identified compounds existing in *N. lappaceum* acetone extract identified by LC-MS analysis

| **No** | **m/z** | **Identified compounds** | **Molecular Formula** | **RT (Min.)** | **Mass** | **Score (DB)** | **Antibacterial activity**  **report** |
| --- | --- | --- | --- | --- | --- | --- | --- |
| 1 | 175.0597 | 6,7-dihydroxy-4-oxo-2-heptenoic acid | C_7_ H_10_ O_5_ | 15.395 | 174.0525 | 98.44 | Not reported |
| 2 | 195.0865 | Quebrachitol | C_7_ H_14_ O_6_ | 1.602 | 194.0792 | 99.65 | 40 |
| 3 | 198.0973 | L-Galactose | C_6_ H_12_ O_6_ | 1.561 | 180.0632 | 94.41 | Not reported |
| 4 | 226.1288 | Ethyl beta-D-glucopyranoside | C_8_ H_16_ O_6_ | 2.208 | 208.0949 | 96.24 | Not reported |
| 5 | 267.1717 | Falcarinol | C_17_ H_24_ O | 18.571 | 244.1825 | 86.46 | 58 |
| 6 | 279.108 | 1-O-(4-hydroxy-2-methylene-butanoyl)-beta-D-glucopyranose | C_11_ H_18_ O_8_ | 15.478 | 278.1009 | 95.32 | Not reported |
| 7 | 279.1556 | Mycosporine | C_11_ H_19_ N O_6_ | 3.478 | 261.1219 | 93.66 | Not reported |
| 8 | 284.1343 | 1-(3-Carboxypropyl)-3,7-dimethylxanthine | C_11_ H_14_ N_4_ O_4_ | 2.814 | 266.1008 | 92.11 | Not reported |
| 9 | 291.0861 | Oritin-4beta-ol | C_15_ H_14_ O_6_ | 10.148 | 290.0789 | 97.34 | Not reported |
| 10 | 303.0144 | Ellagic acid | C_14_ H_6_ O_8_ | 11.041 | 302.0071 | 97.6 | 44 |
| 11 | 369.1334 | Glycyrrhisoflavanone | C_21_ H_20_ O_6_ | 16.226 | 368.1263 | 93.19 | Not reported |
| 12 | 386.1598 | Papaverrubine B | C_21_ H_23_ N O_6_ | 9.801 | 385.1529 | 94.16 | Not reported |
| 13 | 415.2117 | Eplerenone | C_24_ H_30_ O_6_ | 16.629 | 414.2046 | 97.52 | 48 |
| 14 | 420.1858 | Ile His Glu | C_17_ H_27_ N_5_ O_6_ | 9.776 | 397.1966 | 94.94 | Not reported |
| 15 | 430.2437 | 5-(2,3-Dihydroxy-3-methylbutyl)-4-(3,4-epoxy-4-methylpentanoyl)-3,4-dihydroxy-2-isopentanoyl-2-cyclopenten-1-one | C_21_ H_32_ O_8_ | 11.011 | 412.2096 | 92.06 | Not reported |
| 16 | 437.3415 | Demethylphylloquinone | C_30_ H_44_ O_2_ | 15.442 | 436.3342 | 99.08 | Not reported |
| 17 | 439.3573 | Ganoderol A | C_30_ H_46_ O_2_ | 17.197 | 438.35 | 98.68 | Not reported |
| 18 | 449.1077 | 6-Hydroxyluteolin 5-rhamnoside | C_21_ H_20_ O_11_ | 11.364 | 448.1008 | 96.89 | Not reported |
| 19 | 455.352 | Bryononic acid | C_30_ H_46_ O_3_ | 16.158 | 454.3447 | 99.82 | 50 |
| 20 | 465.103 | 8-Hydroxyluteolin 8-glucoside | C_21_ H_20_ O_12_ | 10.901 | 464.0957 | 99.06 | Not reported |
| 21 | 565.3882 | 3beta-Acetoxy-11alpha-methoxy-12-ursen-28-oic acid | C_34_ H_54_ O_5_ | 15.445 | 542.3987 | 93.93 | Not reported |
| 22 | 583.3988 | OH-Diaponeurosporene glucoside ester | C_36_ H_54_ O_6_ | 15.467 | 582.3915 | 95.54 | Not reported |
| 23 | 587.3936 | (3b,16a,21b,22a)-12-Oleanene-3,16,21,23,28-pentol-22-angeloyloxy-23-al | C_35_ H_54_ O_7_ | 16.153 | 586.3866 | 98.4 | Not reported |
| 24 | 593.2756 | Pheophorbide a | C_35_ H_36_ N_4_ O_5_ | 22.885 | 592.2687 | 97.71 | 51 |
| 25 | 611.1397 | Isoorientin 6''-O-caffeate | C_30_ H_26_ O_14_ | 12.087 | 610.1325 | 98.66 | Not reported |
| 26 | 619.4202 | 19-Methoxypomolic acid 3-arabinoside | C_36_ H_58_ O_8_ | 15.472 | 618.4129 | 99.13 | Not reported |
| 27 | 627.3866 | Hederagenin 3-O-arabinoside | C_35_ H_56_ O_8_ | 16.13 | 604.398 | 84.9 | Not reported |
| 28 | 629.4039 | Zizyphine A | C_33_ H_49_ N_5_ O_6_ | 15.349 | 611.3702 | 90.78 | Not reported |
| 29 | 652.1135 | Punicacortein A | C_27_ H_22_ O_18_ | 9.866 | 634.08 | 96.87 | Not reported |
| 30 | 700.2808 | Bruceoside A | C_32_ H_42_ O_16_ | 10.404 | 682.2462 | 92.84 | Not reported |
| 31 | 751.4624 | Oleanolic acid 3-[glucosyl-(1->4)-xyloside] | C_41_ H_66_ O_12_ | 15.441 | 750.4551 | 99.81 | Not reported |
| 32 | 757.4517 | Gambierol | C_43_ H_64_ O_11_ | 15.343 | 756.4438 | 94.87 | Not reported |
| 33 | 775.4617 | Melilotoside B | C_41_ H_68_ O_12_ | 15.382 | 752.4725 | 96.45 | Not reported |
| 34 | 779.4568 | Momordin Ia | C_42_ H_66_ O_13_ | 16.199 | 778.4497 | 99.4 | Not reported |
| 35 | 783.0681 | Emblicanin A | C_34_ H_22_ O_22_ | 9.959 | 782.0606 | 99.34 | 53 |
| 36 | 785.081 | Pedunculagin | C_34_ H_24_ O_22_ | 10.437 | 784.0744 | 91.32 | 54 |
| 37 | 793.4733 | Sanguisorbin E | C_43_ H_68_ O_13_ | 15.448 | 792.4658 | 99.52 | Not reported |
| 38 | 804.1255 | Tercatain | C_34_ H_26_ O_22_ | 10.396 | 786.0909 | 93.46 | 55 |
| 39 | 817.472 | Hebevinoside III | C_43_ H_70_ O_13_ | 16.296 | 794.4827 | 95.75 | Not reported |
| 40 | 865.196 | Epicatechin-(4beta->6)-epicatechin-(2beta->7,4beta->8)-epicatechin | C_45_ H_36_ O_18_ | 10.275 | 864.189 | 92.64 | Not reported |
| 41 | 907.5038 | 3-O-(Rhaa1-4(Rhaa1-2) Glcb) -(25R)-spirost-5-en-3beta-ol | C_46_ H_76_ O_16_ | 15.365 | 884.5145 | 97.89 | Not reported |
| 42 | 970.1154 | Geraniin | C_41_ H_28_ O_27_ | 9.962 | 952.0812 | 99.07 | 57 |
| 43 | 1227.6122 | Eleutheroside M | C_59_ H_96_ O_25_ | 15.357 | 1204.6233 | 97.12 | Not reported |
| 44 | 1231.7826 | Lienomycin | C_67_ H_107_ N O_18_ | 16.127 | 1213.749 | 98.32 | Not reported |

**Table S5.** Virtual Screening and Pharmacological Screening Results

| **MOLECULE** | **VIRTUAL SCREENING** | | | | **PHARMACOLOGICAL SCREENING** | | | | | | | | | | | | | | | | | |
| --- | --- | --- | --- | --- | --- | --- | --- | --- | --- | --- | --- | --- | --- | --- | --- | --- | --- | --- | --- | --- | --- | --- |
|  |  |  |  |  | **ABSORPTION** | | | **METABOLISM** | | | | | |  | **DRUG-LIKENESS** | | | | |  | **Abbot Bioavailability Score** | |
|  | **Binding Energy (kcal/mol)** | | | |  |  |  |  |  |  |  |  |  |  |  |  |  |  |  |  |  |  |
|  | ***P. aeruginosa* DnaK** | ***S. aureus* DnaK** | **Average** | **Standard Deviation** | **GI absorption** | **BBB permeant** | **PGP Substrate** |  | **CYP1A2 inhibitor** | **CYP2C19 inhibitor** | **CYP2C9 inhibitor** | **CYP2D6 inhibitor** | **CYP3A4 inhibitor** |  | **Lipinski #violations** | **Ghose #violations** | **Veber #violations** | **Egan #violations** | **Muegge #violations** |  |  |  |
| Stigmasterol | -8.73 | -10.54 | -9.635 | 0.905 | Low | No | No |  | No | No | Yes | No | No |  | 1 | 3 | 0 | 1 | 2 |  | 0.55 | |
| Demethyl phylloquinone | -9.05 | -9.71 | -9.38 | 0.33 | Low | No | Yes |  | Yes | No | No | No | No |  | 1 | 3 | 1 | 1 | 1 |  | 0.55 | |
| Zizyphine A | -9.49 | -8.59 | -9.04 | 0.45 | High | No | Yes |  | No | No | No | No | Yes |  | 2 | 3 | 1 | 0 | 1 |  | 0.17 | |
| 6-Hydroxyluteolin 5-rhamnoside | -7.35 | -10.64 | -8.995 | 1.645 | Low | No | No |  | No | No | No | No | No |  | 2 | 0 | 1 | 1 | 3 |  | 0.17 | |
| Silane, [[(3alpha,5beta,20S)-pregn-11-ene-3,11,17,20-tetrayl] tetrakis(oxy)] tetrakis [trimethyl] | -9.21 | -8.57 | -8.89 | 0.32 | Low | No | No |  | No | No | No | No | No |  | 2 | 4 | 0 | 1 | 2 |  | 0.17 | |
| Epigallocatechin gallate | -7.68 | -9.76 | -8.72 | 1.04 | Low | No | No |  | No | No | No | No | No |  | 2 | 0 | 1 | 1 | 3 |  | 0.17 | |
| Papaverrubine B | -8.13 | -9.21 | -8.67 | 0.54 | High | Yes | Yes |  | No | No | No | Yes | Yes |  | 0 | 0 | 0 | 0 | 0 |  | 0.55 | |
| OH-Diaponeurosporene glucoside ester | -7.72 | -9.48 | -8.6 | 0.88 | Low | No | Yes |  | No | No | No | No | Yes |  | 1 | 4 | 1 | 1 | 1 |  | 0.55 | |
| Isoorientin 6''-O-caffeate | -8.21 | -8.97 | -8.59 | 0.38 | Low | No | No |  | No | No | No | No | No |  | 3 | 2 | 1 | 1 | 4 |  | 0.17 | |
| Phytosphingosine | -8.73 | -8.33 | -8.53 | 0.2 | High | No | Yes |  | No | No | No | Yes | No |  | 0 | 0 | 1 | 0 | 1 |  | 0.55 | |
| Apigenin 7-(3''-p-coumaroylglucoside) | -8.04 | -8.98 | -8.51 | 0.47 | Low | No | No |  | No | No | Yes | No | No |  | 3 | 2 | 1 | 1 | 3 |  | 0.17 | |
| Aurmillone | -8.07 | -8.94 | -8.505 | 0.435 | High | No | No |  | No | No | Yes | No | Yes |  | 0 | 0 | 0 | 0 | 0 |  | 0.55 | |
| Ganoderol A | -8.8 | -8.09 | -8.445 | 0.355 | Low | No | No |  | No | No | No | No | No |  | 1 | 3 | 0 | 1 | 1 |  | 0.55 | |
| Robinetin 3-rutinoside | -8.04 | -8.83 | -8.435 | 0.395 | Low | No | Yes |  | No | No | No | No | No |  | 3 | 4 | 1 | 1 | 4 |  | 0.17 | |
| Alpha-Tocopherol | -7.91 | -8.68 | -8.295 | 0.385 | Low | No | Yes |  | No | No | No | No | No |  | 1 | 3 | 1 | 1 | 1 |  | 0.55 | |
| 2,2-Bis[4-[(4,6-dichloro-1,3,5-triazin-2-yl) oxy] phenyl]-1,1,1,3,3,3-hexafluoropropane | -8.48 | -8.06 | -8.27 | 0.21 | Low | No | Yes |  | Yes | No | No | No | No |  | 2 | 2 | 0 | 1 | 3 |  | 0.17 | |
| **Catechin** | **-7.83** | **-8.58** | **-8.205** | **0.375** | **High** | **No** | **Yes** |  | **No** | **No** | **No** | **No** | **No** |  | **0** | **0** | **0** | **0** | **0** |  | **0.55** | |
| Quercetin | -8.17 | -8.16 | -8.165 | 0.005 | High | No | No |  | Yes | No | No | Yes | Yes |  | 0 | 0 | 0 | 0 | 0 |  | 0.55 | |
| 8-Hydroxyluteolin 8-glucoside | -6.39 | -9.83 | -8.11 | 1.72 | Low | No | No |  | No | No | No | No | No |  | 2 | 1 | 1 | 1 | 3 |  | 0.17 | |
| (4OH,8Z, t18:1) sphingosine | -8.16 | -7.89 | -8.025 | 0.135 | High | No | Yes |  | No | No | No | Yes | No |  | 0 | 0 | 1 | 0 | 0 |  | 0.55 | |
| Bryononic acid | -6.87 | -9.18 | -8.025 | 1.155 | Low | No | No |  | No | No | No | No | No |  | 1 | 3 | 0 | 1 | 1 |  | 0.85 | |
| **Eplerenone** | **-7.8** | **-8.16** | **-7.98** | **0.18** | **High** | **No** | **No** |  | **No** | **No** | **No** | **No** | **No** |  | **0** | **0** | **0** | **0** | **0** |  | **0.55** | |
| Card-20(22)-enolide, 3-[(6-deoxy-3,4-O-methylenehexopyranos-2-ulos-1-yl)oxy]-5,11,14-trihydroxy-12-oxo-, (3.beta.,5.alpha.,11.alpha.)- | -8.78 | -7.11 | -7.945 | 0.835 | Low | No | Yes |  | No | No | No | No | No |  | 2 | 3 | 1 | 1 | 2 |  | 0.17 | |
| beta-Hederin | -7.54 | -8.31 | -7.925 | 0.385 | Low | No | Yes |  | No | No | No | No | No |  | 3 | 3 | 1 | 1 | 4 |  | 0.11 | |
| Glycyrrhisoflavanone | -7.67 | -7.9 | -7.785 | 0.115 | High | No | Yes |  | Yes | Yes | Yes | Yes | Yes |  | 0 | 0 | 0 | 0 | 0 |  | 0.55 | |
| Avicularin | -6.49 | -9.02 | -7.755 | 1.265 | Low | No | No |  | No | No | No | No | No |  | 2 | 0 | 1 | 1 | 3 |  | 0.17 | |
| 2'-Hydroxyfurano [2'',3'':4',3’] chalcone | -7.63 | -7.72 | -7.675 | 0.045 | High | Yes | No |  | Yes | Yes | Yes | No | No |  | 0 | 0 | 0 | 0 | 0 |  | 0.55 | |
| Chlorogenic acid | -7.85 | -7.47 | -7.66 | 0.19 | Low | No | No |  | No | No | No | No | No |  | 1 | 1 | 1 | 1 | 2 |  | 0.11 | |
| (-)-Glyceollin I | -7.51 | -7.7 | -7.605 | 0.095 | High | Yes | Yes |  | Yes | Yes | No | Yes | Yes |  | 0 | 0 | 0 | 0 | 0 |  | 0.55 | |
| 5H-Cyclopropa (3,4) benz(1,2-e) azulen-5-one, 1,1a-à,1b-á,4,4a,7a-à,7b,8,9,9a-decahydro-7b-à,9-á,9a-à-trihydroxy-3-hydroxymethyl-1,1,6,8-à-tetramethyl-4a-methoxy-, 9,9a-didecanoate | -7.77 | -7.22 | -7.495 | 0.275 | Low | No | Yes |  | No | No | No | No | Yes |  | 2 | 4 | 1 | 1 | 3 |  | 0.17 | |
| Kinetin Riboside | -6.8 | -8.05 | -7.425 | 0.625 | Low | No | No |  | No | No | No | No | No |  | 0 | 1 | 0 | 1 | 0 |  | 0.55 | |
| Ellagic acid | -6.68 | -8.14 | -7.41 | 0.73 | High | No | No |  | Yes | No | No | No | No |  | 0 | 0 | 1 | 1 | 0 |  | 0.55 | |
| Curlone | -7.11 | -7.57 | -7.34 | 0.23 | High | Yes | No |  | No | Yes | Yes | No | No |  | 0 | 0 | 0 | 0 | 1 |  | 0.55 | |
| Mycosporine | -7.78 | -6.87 | -7.325 | 0.455 | Low | No | No |  | No | No | No | No | No |  | 0 | 1 | 0 | 0 | 1 |  | 0.55 | |
| 5-(2,3-Dihydroxy-3-methylbutyl)-4-(3,4-epoxy-4-methylpentanoyl)-3,4-dihydroxy-2-isopentanoyl-2-cyclopenten-1-one | -6.95 | -7.63 | -7.29 | 0.34 | Low | No | Yes |  | No | No | No | No | No |  | 0 | 0 | 1 | 1 | 0 |  | 0.56 | |
| **Oritin-4beta-ol** | **-7.08** | **-7.3** | **-7.19** | **0.11** | **High** | **No** | **No** |  | **No** | **No** | **No** | **No** | **No** |  | **0** | **0** | **0** | **0** | **0** |  | **0.55** | |
| (3b,16a,21b,22a)-12-Oleanene-3,16,21,23,28-pentol-22-angeloyloxy-23-al | -6.29 | -8.01 | -7.15 | 0.86 | Low | No | Yes |  | No | No | No | No | Yes |  | 1 | 3 | 0 | 0 | 1 |  | 0.55 | |
| Momordin Ia | -7.17 | -7.1 | -7.135 | 0.035 | Low | No | Yes |  | No | No | No | No | No |  | 3 | 3 | 1 | 1 | 4 |  | 0.11 | |
| 1,3,4,5-Tetra-O-galloylquinic acid | -5.96 | -8.25 | -7.105 | 1.145 | Low | No | Yes |  | No | No | No | No | No |  | 3 | 3 | 2 | 1 | 4 |  | 0.11 | |
| 5,6-Dihydrouridine | -6.71 | -7.38 | -7.045 | 0.335 | Low | No | No |  | No | No | No | No | No |  | 0 | 1 | 0 | 0 | 1 |  | 0.55 | |
| Ar-tumerone | -6.7 | -7.23 | -6.965 | 0.265 | High | Yes | No |  | No | No | No | No | No |  | 0 | 0 | 0 | 0 | 1 |  | 0.55 | |
| Phenol, 2,4-bis(1,1-dimethylethyl) | -6.89 | -6.75 | -6.82 | 0.07 | High | Yes | No |  | No | No | No | Yes | No |  | 0 | 0 | 0 | 0 | 2 |  | 0.55 | |
| 3,7,11-Tridecatrienenitrile, 4,8,12-trimethyl | -6.49 | -7.05 | -6.77 | 0.28 | High | Yes | No |  | Yes | No | Yes | No | No |  | 0 | 0 | 0 | 0 | 2 |  | 0.55 | |
| Benzyl O-[arabinofuranosyl-(1->6)-glucoside] | -5.85 | -7.64 | -6.745 | 0.895 | Low | No | No |  | No | No | No | No | No |  | 1 | 1 | 1 | 1 | 3 |  | 0.55 | |
| Ile His Glu | -5.35 | -8.03 | -6.69 | 1.34 | Low | No | No |  | No | No | No | No | No |  | 2 | 1 | 2 | 1 | 3 |  | 0.17 | |
| 1-O-(4-hydroxy-2-methylene-butanoyl)-beta-D-glucopyranose | -6.77 | -6.57 | -6.67 | 0.1 | Low | No | Yes |  | No | No | No | No | No |  | 0 | 1 | 0 | 1 | 0 |  | 0.55 | |
| Sanguisorbin E | -7.98 | -5.17 | -6.575 | 1.405 | Low | No | Yes |  | No | No | No | No | No |  | 3 | 3 | 1 | 1 | 4 |  | 0.17 | |
| Pheophorbide a | -6.98 | -6.02 | -6.5 | 0.48 | Low | No | Yes |  | No | No | Yes | No | No |  | 1 | 3 | 0 | 1 | 0 |  | 0.56 | |
| 19-Methoxypomolic acid 3-arabinoside | -6.2 | -6.68 | -6.44 | 0.24 | Low | No | Yes |  | No | No | No | No | No |  | 1 | 3 | 0 | 0 | 1 |  | 0.55 | |
| Emblicanin A | -6.74 | -6.13 | -6.435 | 0.305 | Low | No | Yes |  | No | No | No | No | No |  | 3 | 3 | 1 | 1 | 4 |  | 0.11 | |
| Melilotoside B | -4.98 | -7.83 | -6.405 | 1.425 | Low | No | Yes |  | No | No | No | No | No |  | 3 | 3 | 1 | 1 | 4 |  | 0.17 | |
| 3beta-Acetoxy-11alpha-methoxy-12-ursen-28-oic acid | -6.71 | -6.1 | -6.405 | 0.305 | Low | No | No |  | No | No | No | No | No |  | 2 | 4 | 0 | 1 | 1 |  | 0.17 | |
| 2-Amino-3-methyl-1-butanol | -6.42 | -6.37 | -6.395 | 0.025 | High | No | No |  | No | No | No | No | No |  | 0 | 2 | 0 | 0 | 1 |  | | 0.55 |
| Gambierol | -7.19 | -5.46 | -6.325 | 0.865 | Low | No | Yes |  | No | No | No | No | No |  | 2 | 3 | 0 | 1 | 3 |  | | 0.17 |
| 1-(3-Carboxypropyl)-3,7-dimethylxanthine | -6.16 | -6.31 | -6.235 | 0.075 | High | No | No |  | No | No | No | No | No |  | 0 | 1 | 0 | 0 | 0 |  | | 0.56 |
| 2,3-Butanediol glucoside | -6.67 | -5.72 | -6.195 | 0.475 | Low | No | Yes |  | No | No | No | No | No |  | 0 | 1 | 0 | 0 | 1 |  | | 0.55 |
| Tromethamine | -5.97 | -6.42 | -6.195 | 0.225 | Low | No | No |  | No | No | No | No | No |  | 0 | 4 | 0 | 0 | 3 |  | | 0.55 |
| Bruceoside A | -6.28 | -6 | -6.14 | 0.14 | Low | No | No |  | No | No | No | No | No |  | 3 | 4 | 1 | 1 | 4 |  | | 0.17 |
| Tercatain | -6.64 | -5.6 | -6.12 | 0.52 | Low | No | Yes |  | No | No | No | No | No |  | 3 | 3 | 1 | 1 | 4 |  | | 0.17 |
| 1,3-Dioxolane, 2-pentadecyl | -6.08 | -6.09 | -6.085 | 0.005 | High | No | No |  | Yes | No | No | No | No |  | 0 | 1 | 1 | 0 | 1 |  | | 0.55 |
| Falcarinol | -6.27 | -5.84 | -6.055 | 0.215 | High | Yes | No |  | Yes | No | Yes | No | No |  | 1 | 0 | 0 | 0 | 2 |  | | 0.55 |
| Vitamin C | -6.31 | -5.78 | -6.045 | 0.265 | High | No | No |  | No | No | No | No | No |  | 0 | 2 | 0 | 0 | 1 |  | | 0.56 |
| Iretol | -6.51 | -5.54 | -6.025 | 0.485 | High | No | No |  | No | No | No | No | Yes |  | 0 | 3 | 0 | 0 | 1 |  | | 0.55 |
| L-Galactose | -6.38 | -5.51 | -5.945 | 0.435 | Low | No | Yes |  | No | No | No | No | No |  | 0 | 2 | 0 | 0 | 2 |  | | 0.55 |
| Hederagenin 3-O-arabinoside | -6.74 | -5.1 | -5.92 | 0.82 | Low | No | Yes |  | No | No | No | No | No |  | 1 | 3 | 0 | 1 | 1 |  | | 0.56 |
| Salicyl phenolic glucuronide | -6.07 | -5.76 | -5.915 | 0.155 | Low | No | No |  | No | No | No | No | No |  | 0 | 1 | 1 | 1 | 1 |  | | 0.11 |
| 3-O-(Rhaa1-4(Rhaa1-2) Glcb) -(25R)-spirost-5-en-3beta-ol | -5.49 | -6.32 | -5.905 | 0.415 | Low | No | Yes |  | No | No | No | No | No |  | 3 | 3 | 1 | 1 | 5 |  | | 0.17 |
| Niflumic Acid | -5.99 | -5.71 | -5.85 | 0.14 | High | Yes | No |  | No | No | No | No | No |  | 0 | 0 | 0 | 0 | 0 |  | | 0.85 |
| n-Hexadecanoic acid | -5.86 | -5.63 | -5.745 | 0.115 | High | Yes | No |  | Yes | No | Yes | No | No |  | 1 | 0 | 1 | 0 | 1 |  | | 0.85 |
| Hebevinoside VI | -6.24 | -5.23 | -5.735 | 0.505 | Low | No | Yes |  | No | No | No | No | No |  | 3 | 3 | 1 | 1 | 4 |  | | 0.17 |
| Ethyl beta-D-glucopyranoside | -6.02 | -5.4 | -5.71 | 0.31 | High | No | Yes |  | No | No | No | No | No |  | 0 | 1 | 0 | 0 | 1 |  | | 0.55 |
| Phenol, 2-methoxy-3-(2-propenyl) | -5.87 | -5.52 | -5.695 | 0.175 | High | Yes | No |  | Yes | No | No | No | No |  | 0 | 0 | 0 | 0 | 1 |  | | 0.55 |
| Hebevinoside III | -7.02 | -4.28 | -5.65 | 1.37 | Low | No | Yes |  | No | No | No | No | No |  | 3 | 3 | 2 | 1 | 4 |  | | 0.17 |
| Quebrachitol | -5.98 | -5.19 | -5.585 | 0.395 | Low | No | Yes |  | No | No | No | No | No |  | 0 | 1 | 0 | 0 | 2 |  | | 0.55 |
| Oleanolic acid 3-[glucosyl-(1->4)-xyloside] | -6.79 | -4.37 | -5.58 | 1.21 | Low | No | Yes |  | No | No | No | No | No |  | 3 | 3 | 1 | 1 | 4 |  | | 0.11 |
| Punicacortein A | -5.82 | -4.94 | -5.38 | 0.44 | Low | No | Yes |  | No | No | No | No | No |  | 3 | 3 | 1 | 1 | 4 |  | | 0.17 |
| Reduced flavine adenine dinucleotide (FADH2) | -4 | -6.59 | -5.295 | 1.295 | Low | No | Yes |  | No | No | No | No | No |  | 3 | 4 | 2 | 1 | 5 |  | | 0.11 |
| Epicatechin-(4beta->6)-epicatechin-(2beta->7,4beta->8)-epicatechin | -5.4 | -5.14 | -5.27 | 0.13 | Low | No | Yes |  | No | No | Yes | No | No |  | 3 | 3 | 1 | 1 | 5 |  | | 0.17 |
| 6,7-dihydroxy-4-oxo-2-heptenoic acid | -5.04 | -5.14 | -5.09 | 0.05 | High | No | No |  | No | No | No | No | No |  | 0 | 2 | 0 | 0 | 1 |  | | 0.56 |
| Pitheduloside B | -6.63 | -3.47 | -5.05 | 1.58 | Low | No | Yes |  | No | No | No | No | No |  | 3 | 3 | 1 | 1 | 5 |  | | 0.11 |
| p-Salicylic acid | -5.04 | -4.98 | -5.01 | 0.03 | High | Yes | No |  | No | No | No | No | No |  | 0 | 3 | 0 | 0 | 1 |  | | 0.85 |
| Caffeine | -4.58 | -4.66 | -4.62 | 0.04 | High | No | No |  | No | No | No | No | No |  | 0 | 1 | 0 | 0 | 1 |  | | 0.55 |
| 2-Methoxy-1,3-dioxolane | -4.93 | -4.31 | -4.62 | 0.31 | High | No | No |  | No | No | No | No | No |  | 0 | 3 | 0 | 0 | 2 |  | | 0.55 |
| D-Glucoside | -4.59 | -4.52 | -4.555 | 0.035 | Low | No | Yes |  | No | No | No | No | Yes |  | 2 | 3 | 1 | 1 | 3 |  | | 0.11 |
| Geraniin | -6.46 | -2.11 | -4.285 | 2.175 | Low | No | Yes |  | No | No | No | No | No |  | 3 | 4 | 1 | 1 | 5 |  | | 0.17 |
| Malic acid | -3.97 | -3.91 | -3.94 | 0.03 | High | No | No |  | No | No | No | No | No |  | 0 | 4 | 0 | 0 | 2 |  | | 0.56 |
| 3-Methyl-1,2-diazirine | -3.6 | -3.43 | -3.515 | 0.085 | Low | No | No |  | No | No | No | No | No |  | 0 | 4 | 0 | 0 | 2 |  | | 0.55 |
| Pedunculagin | -3.74 | -1.05 | -2.395 | 1.345 | Low | No | Yes |  | No | No | No | No | No |  | 3 | 3 | 1 | 1 | 4 |  | | 0.17 |
| Chebulagic acid | -3.85 | 5.52 | 0.835 | 4.685 | Low | No | Yes |  | No | No | No | No | No |  | 3 | 3 | 1 | 1 | 5 |  | | 0.11 |
| Eleutheroside M | 2.18 | 12.62 | 7.4 | 5.22 | Low | No | Yes |  | No | No | No | No | No |  | 3 | 4 | 2 | 1 | 5 |  | | 0.17 |
| Lienomycin | 201.78 | 206.25 | 204.015 | 2.235 | Low | No | Yes |  | No | No | No | No | No |  | 3 | 3 | 2 | 1 | 5 |  | | 0.17 |
|  |  |  |  |  |  |  |  |  |  |  |  |  |  |  |  |  |  |  |  |  | |  |

**Note.** #violations refer to number of violations in drug-likeness rules. The predicted pharmacological properties were colour coded, from green (preferred) to red (unfavoured).

**LC-MS data of Metlin-Unmatched Compounds from *N. lappaceum* Sequential Extract.**

**Table S6.** Molecular Formula of Metlin-Unmatched Compounds from *N. lappaceum* Ethyl Acetate Extract.

| **NO** | **Molecular Formula** | **RT (Mint)** | **m/z** | **Mass** |
| --- | --- | --- | --- | --- |
|  | C_8_ H_4_ O_3_ | 19.801 | 149.0234 | 148.0161 |
|  | C_9_ H_19_ N O | 15.301 | 158.1536 | 157.1463 |
|  | C_6_ H_2_ N_2_ O_3_ S | 1.361 | 182.9851 | 181.9787 |
|  | C_6_ H_8_ N_3_ O_5_ | 1.541 | 203.0525 | 202.0455 |
|  | C_5_ H_8_ N_6_ O_4_ | 1.582 | 217.068 | 216.0608 |
|  | C_10_ H_8_ N_4_ O_2_ | 16.292 | 217.0717 | 216.0645 |
|  | C_13_ H_25_ N O_2_ | 14.934 | 228.1957 | 227.1879 |
|  | C_9_ H_11_ Cl N O_4_ | 1.761 | 233.0457 | 232.038 |
|  | C_16_ H_34_ O_3_ | 21.544 | 275.2581 | 274.2508 |
|  | C_17_ H_36_ O_3_ | 22.188 | 289.2739 | 288.2662 |
|  | C_13_ H_18_ O_8_ | 15.353 | 303.1075 | 302.1 |
|  | C_18_ H_38_ O_3_ | 23.093 | 303.2898 | 302.2822 |
|  | C_13_ H_20_ O_9_ | 15.481 | 321.1182 | 320.1109 |
|  | C_13_ H_37_ N_9_ O | 21.458 | 336.3184 | 335.3114 |
|  | C_19_ H_40_ O_4_ | 22.189 | 350.3266 | 332.2926 |
|  | C_22_ H_41_ N _O_2 | 21.887 | 352.3236 | 351.316 |
|  | C_21_ H_40_ O_2_ S | 20.935 | 357.283 | 356.2756 |
|  | C_18_ H_43_ N_4_ O_3_ | 23.131 | 364.3421 | 363.3343 |
|  | C_10_ H_22_ N_9_ O_4_ S_2_ | 1.563 | 397.1318 | 396.1235 |
|  | C_19_ H_14_ O_12_ | 10.481 | 435.056 | 434.0489 |
|  | C_25_ H_51_ N_4_ O_2_ | 18.669 | 440.4094 | 439.4018 |
|  | C_29_ H_22_ N_2_ S_2_ | 21.043 | 463.1321 | 462.1229 |
|  | C_21_ H_20_ O_8_ S_2_ | 9.874 | 465.0662 | 464.0601 |
|  | C_27_ H_55_ N_4_ O_2_ | 20.443 | 468.4415 | 467.4315 |
|  | C_20_ H_16_ O_14_ | 9.698 | 481.0608 | 480.0541 |
|  | C_32_ H_28_ N_2_ S_3_ | 22.245 | 554.1757 | 536.1411 |
|  | C_40_ H_17_ N_3_ | 22.245 | 557.1766 | 539.1411 |
|  | C_21_ H_24_ N_10_ S_5_ | 1.736 | 577.085 | 576.0787 |
|  | C_36_ H_56_ O_7_ | 15.47 | 601.4096 | 600.4023 |
|  | C_35_ H_37_ N_3_ S_4_ | 23.432 | 628.1946 | 627.1868 |
|  | C_31_ H_15_ N_15_ O | 23.433 | 631.1941 | 613.1587 |
|  | C_32_ H_52_ N_15_ O | 24.263 | 663.4548 | 662.4478 |
|  | C_26_ H_19_ N_8_ O_14_ | 9.671 | 668.1093 | 667.102 |
|  | C_37_ H_39_ N_3_ S_5_ | 24.274 | 686.1835 | 685.175 |
|  | C_40_ H_56_ N_7_ O_5_ | 15.442 | 715.4415 | 714.4343 |

**Table S7.** Molecular Formula of Metlin-Unmatrched Compounds from *N. lappaceum* Acetone Extract.

| **NO** | **Molecular Formula** | **RT (Mint)** | **m/z** | **Mass** |
| --- | --- | --- | --- | --- |
|  | C_8_ H_4_ O_3_ | 19.8 | 149.0232 | 148.0159 |
|  | C_9_ H_19_ N O | 15.304 | 158.1536 | 157.1463 |
|  | C_11_ H Cl N | 1.363 | 182.9848 | 181.979 |
|  | C_6_ H_8_ N_3_ O_5_ | 1.549 | 203.0526 | 202.0458 |
|  | C_7_ H_4_ N_4_ O_3_ | 2.235 | 210.0627 | 192.0284 |
|  | C_10_ H_7_ N_5_ O_3_ | 1.639 | 246.0613 | 245.0548 |
|  | C_16_ H_34_ O_3_ | 21.538 | 275.2587 | 274.2511 |
|  | C_17_ H_36_ O_3_ | 22.165 | 289.2743 | 288.2666 |
|  | C_23_ H_20_ N_12_ O_8_ | 1.554 | 297.0845 | 592.1539 |
|  | C_18_ H_38_ O_3_ | 23.078 | 303.2896 | 302.2817 |
|  | C_14_ H_16_ N_4_ O_5_ | 15.473 | 321.1189 | 320.1116 |
|  | C_19_ H_40_ O_4_ | 22.17 | 350.3267 | 332.2924 |
|  | C_21_ H_40_ O_2_ S | 20.937 | 357.282 | 356.2749 |
|  | C_20_ H_20_ N O_2_ S_2_ | 22.24 | 371.102 | 370.0942 |
|  | C_10_ H_18_ N_6_ O_10_ | 1.535 | 383.1152 | 382.108 |
|  | C_11_ H_20_ N_6_ O_10_ | 1.579 | 397.1316 | 396.1243 |
|  | C_12_ H_20_ N_13_ S_2_ | 1.612 | 411.1479 | 410.1404 |
|  | C_19_ H_14_ O_12_ | 10.478 | 435.0565 | 434.0492 |
|  | C_17_ H_22_ O_12_ | 9.408 | 436.1455 | 418.1116 |
|  | C_22_ H_16_ N_4_ O_4_ S_2_ | 9.864 | 465.0672 | 464.0608 |
|  | C_20_ H_16_ O_14_ | 9.695 | 481.0615 | 480.0546 |
|  | C_32_ H_28_ N_2_ S_3_ | 22.24 | 554.1762 | 536.1417 |
|  | C_36_ H_56_ O_7_ | 15.451 | 601.4104 | 600.403 |
|  | C_37_ H_39_ O S_4_ | 23.43 | 628.1959 | 627.1878 |
|  | C_27_ H_25_ N O_19_ | 9.689 | 668.109 | 667.102 |
|  | C_30_ H_29_ N O_19_ | 10.177 | 708.1399 | 707.1328 |

**Table S8.** Antibiotic susceptibility profile of bacterial strains used in this study

| **N0** | **Drugs** | **Conc (µg)** | **Bacterial strains** | | | | | |
| --- | --- | --- | --- | --- | --- | --- | --- | --- |
|  |  |  | *B. subtilis* | MRSA | *S. pyogenes* | *P. aeruginosa* | *K. pneumonia* | *S. enterica* |
| 1 | Amoxicillin | 10 | R | R | R | R | R | R |
| 2 | Ampicillin | 10 | R | R | R | R | R | R |
| 3 | Ciprofloxacin | 10 | R | R | S | S | S | S |
| 4 | Colistin | 10 | R | R | I | S | R | S |
| 5 | Erythromycin | 15 | I | R | R | R | R | R |
| 6 | Gentamicin | 10 | S | S | S | S | S | S |
| 7 | Kanamycin | 30 | I | S | R | R | I | R |
| 8 | Norfloxacin | 10 | I | R | S | S | R | R |
| 9 | Tetracycline | 30 | I | S | R | R | R | S |
| 10 | Trimethoprim | 5 | R | S | I | R | S | S |

S: Susceptible, R: Resistant, I: intermediate

**Table S9.** Druggable Pockets Predicted via P2Rank

| **Protein** | **Score** | **Residues** |
| --- | --- | --- |
| *P. aeruginosa* DnaK | 33.6991 | 8, 10, 11, 12, 13, 37, 39, 55, 58, 65, 67, 68, 70, 171, 194, 196, 197, 199, 229, 230, 233, 260, 263, 264, 267, 270, 271, 274, 341, 342, 343, 345, 367, 368, 371 |
| *S. aureus* DnaK | 32.7680 | 8, 10, 11, 12, 13, 14, 15, 35, 145, 168, 170, 171, 173, 199, 200, 237, 240, 241, 311, 312, 313, 315, 337, 338 |


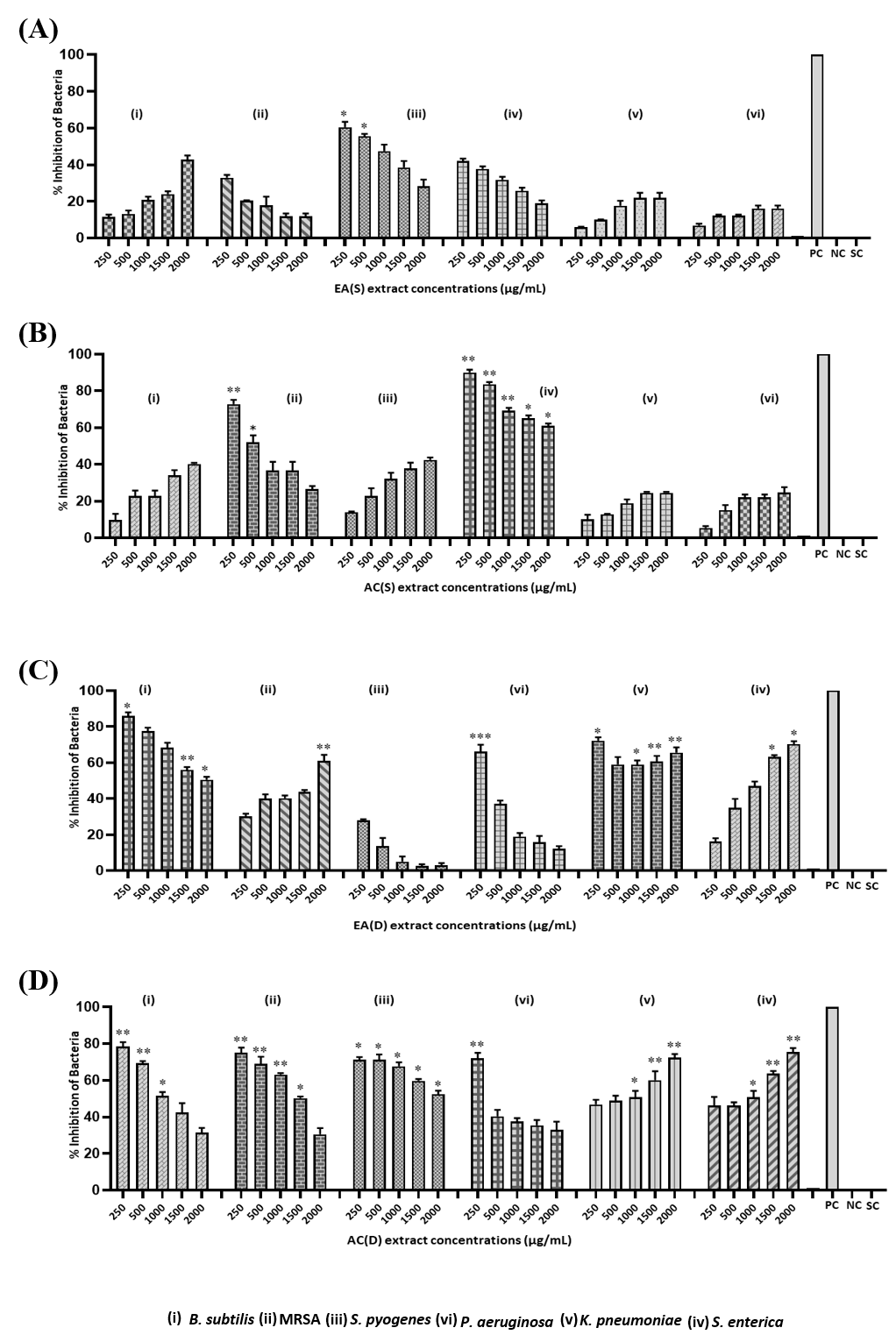
**Potential *in-vitro* antibacterial activities of yellow rambutan fruit epicarp extracts.**

**Figure S1.** Broth dilution method was used for evaluating the antibacterial potential of *N. lappaceum* **(A)** ethyl acetate sequential extract **(B)** acetone sequential **(C)** ethyl acetate direct extract **(D)** acetone direct extract, calculating the inhibition percentage and MIC value of every TP. Briefly, 5 × 10^5^ bacteria were treated using various concentrations (250-2000µg/mL) and the activity was recorded after 16 hours incubation at 37 ^0^C. The data is expressed as the mean ±standard error of several independent experiments performed in technical triplicates. P values were determined using student’s T-test, two-tailed distribution, (*) is P ≤ 0.05. Dimethyl sulfoxide (DMSO), gentamicin (10 µg/mL) and bacteria alone were taken as solvent, positive and negative controls respectively. (i) Extracts tested against *B. subtilis*, (ii) against MRSA, (iii) against *S. pyogenes*, (iv) against *P. aeruginosa,* (v) against *K. pneumonia,* (vi) against *S. enterica*

**HPLC analysis of *N. lappaceum* sequential extracts:**

**
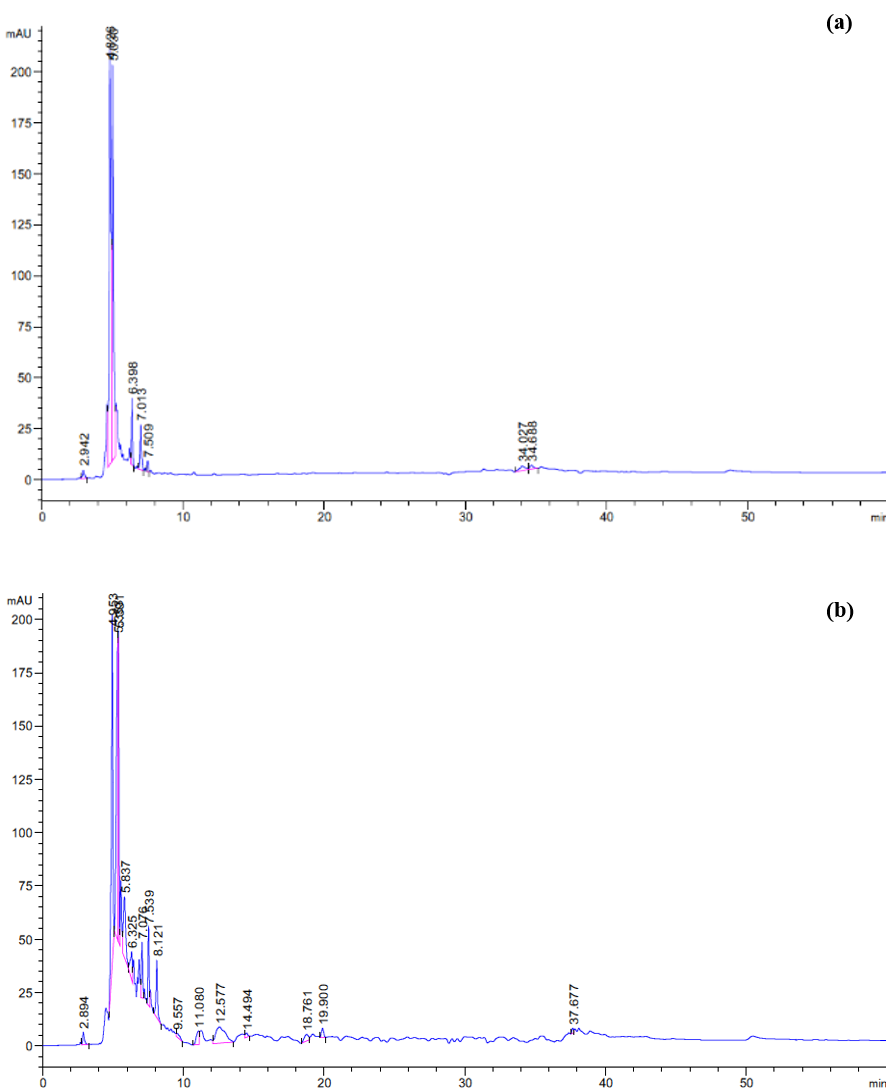
**

**Figure S2.** HPLC-UV chromatogram of sequential fraction for **(a)** ethyl acetate **(b)** acetone.

**
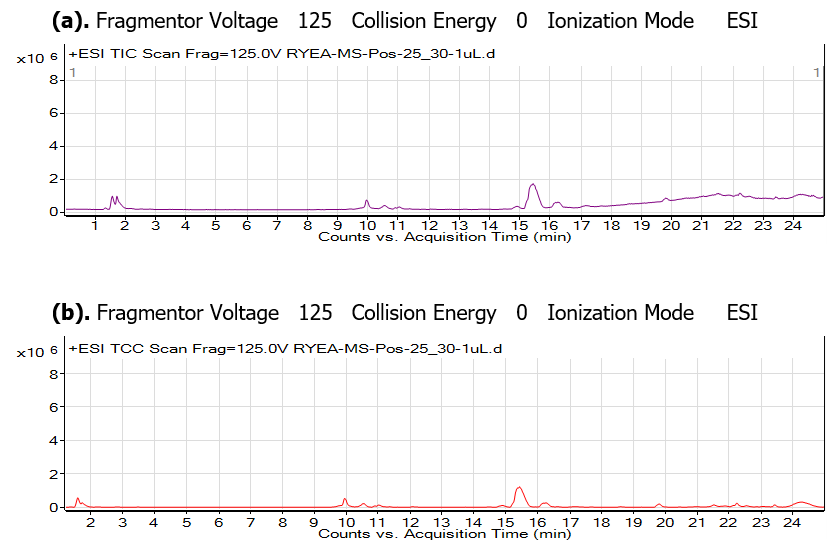
LC-MS analysis of *N. lappaceum* sequential extracts:**

**Figure S3.** *N. lappaceum* ethyl acetate extract was subjected to LCMS qualitative analysis using positive ion mode. **a)** Total ion current (TIC) chromatogram and **b)** Total compound chromatogram (TCC).


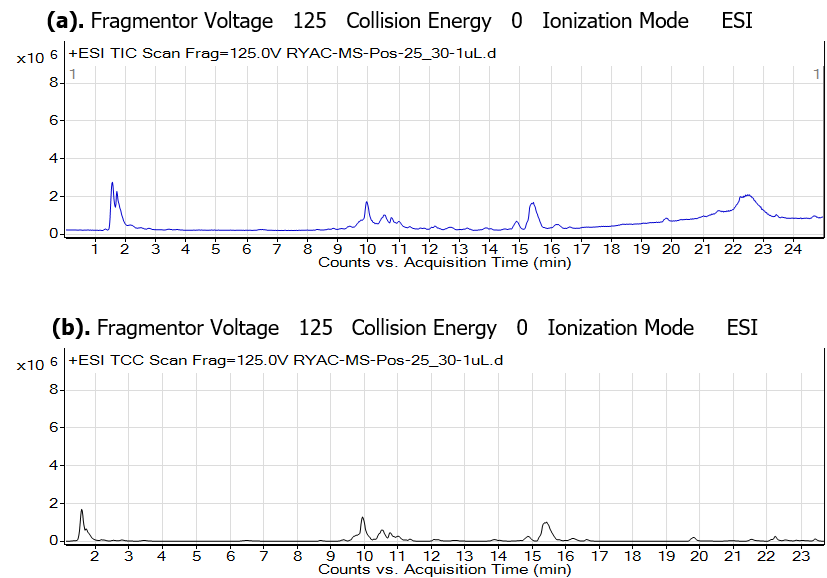


**Figure S4.** *N. lappaceum* acetone extract was subjected to LCMS qualitative analysis using positive ion mode. a) Total ion current (TIC) chromatogram and b) Total compound chromatogram (TCC).

**GC-MS analysis of *N. lappaceum* sequential and direct extracts:**

**
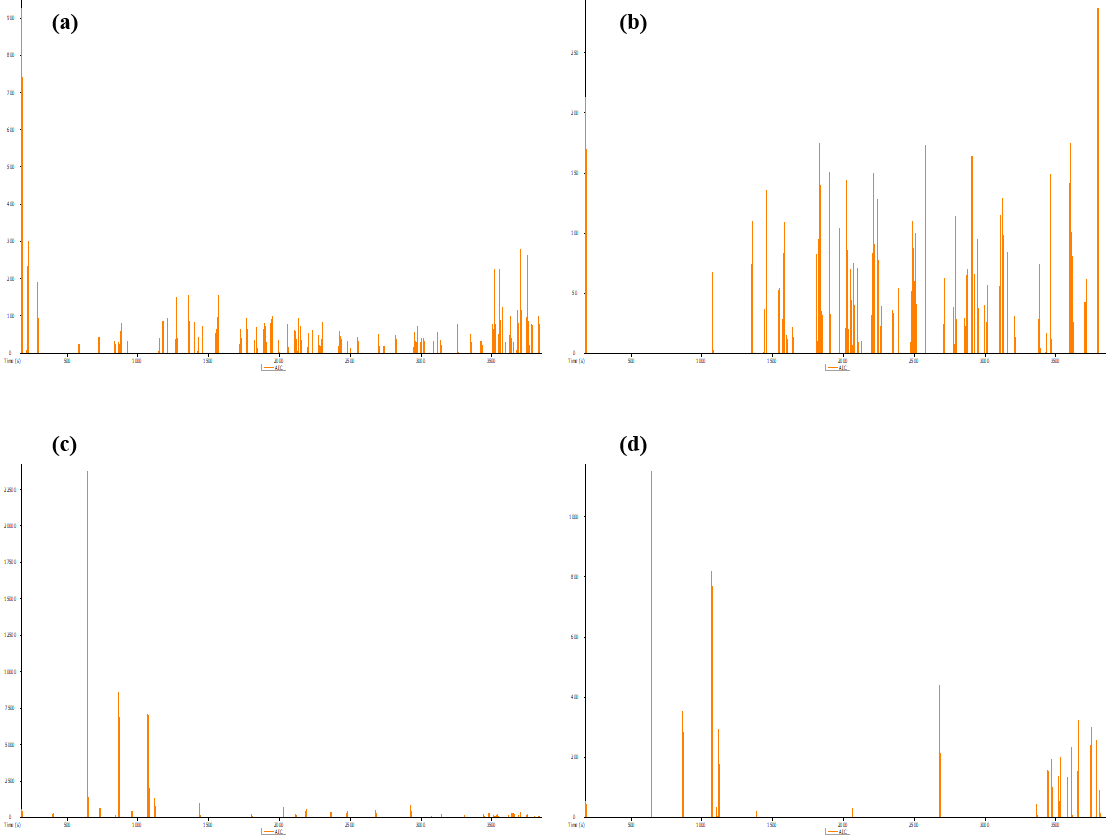
**

**Figure S5:** GC-MS chromatogram of sequential and direct extracts of *N. lappaceum*. Compounds were identified based on % peak area. **(a)** sequential ethyl acetate fraction. **(b)** sequential acetone fraction. **(c)** direct ethyl acetate fraction. **(b)** direct acetone fraction.

**Homology Model Validation**

| 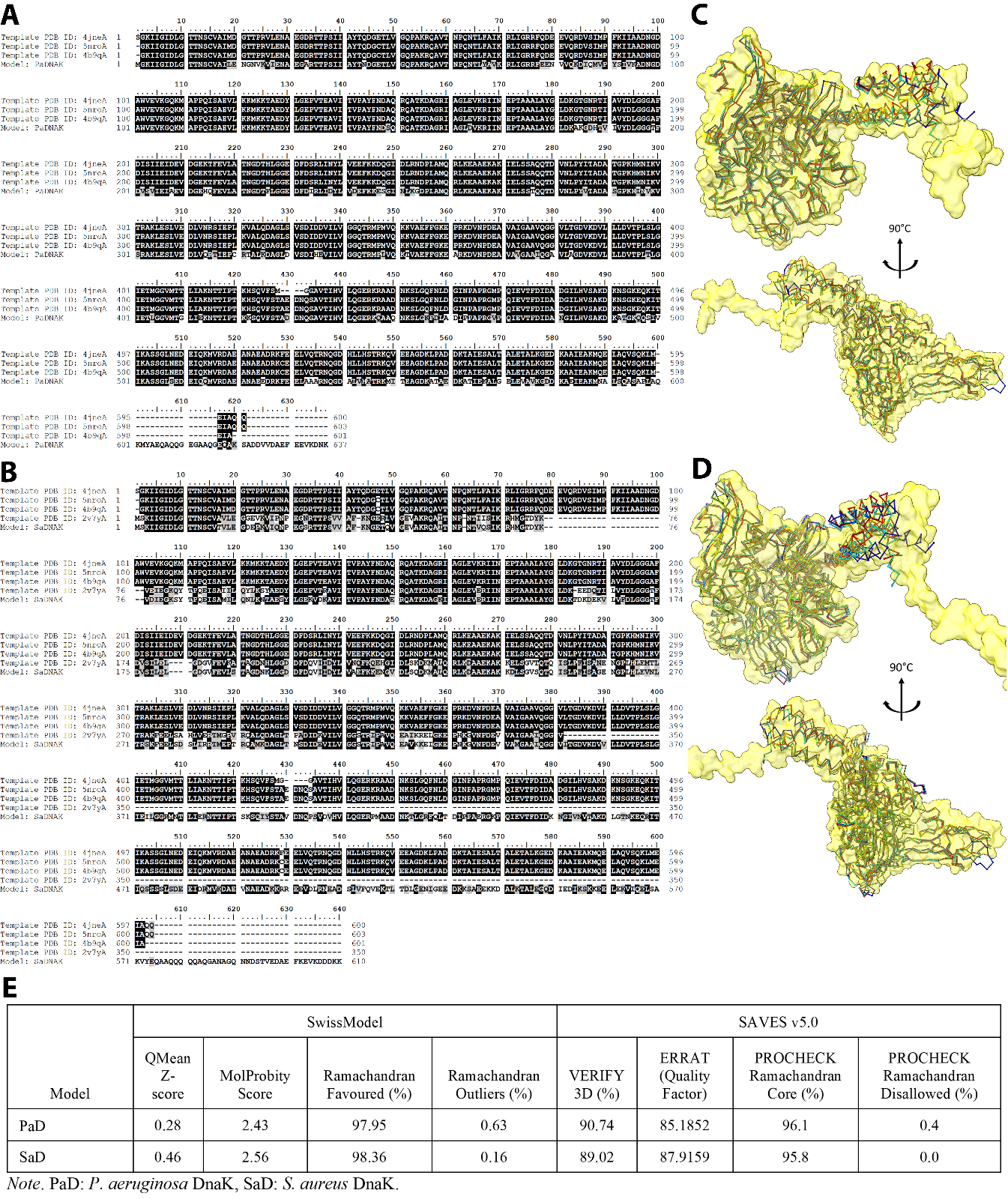 |
| --- |
| **Figure S6**. Validation of *P. aeruginosa* DnaK and *S. aureus* DnaK homology models generated via MODELLER. Sequence alignment between model and templates for **A.** *P. aeruginosa* and **B.** *S. aureus* DnaK were carried out via the MODELLER built-in sequence aligner, as well as visualized using BioEdit^96^. The homology models (yellow surface) of **C.** *P. aeruginosa* and **D.** *S. aureus* DnaK were structurally aligned with respective templates (chain trace), with PDB ID of 4JNE (blue), 5NRO (cyan), 4B9Q (red), and 2V7Y (green). **E**. Quality evaluation of homology models were conducted via the SWISSMODEL and SAVES v5.0 webservers. |

**Druggable Pocket Prediction**

| **A.**  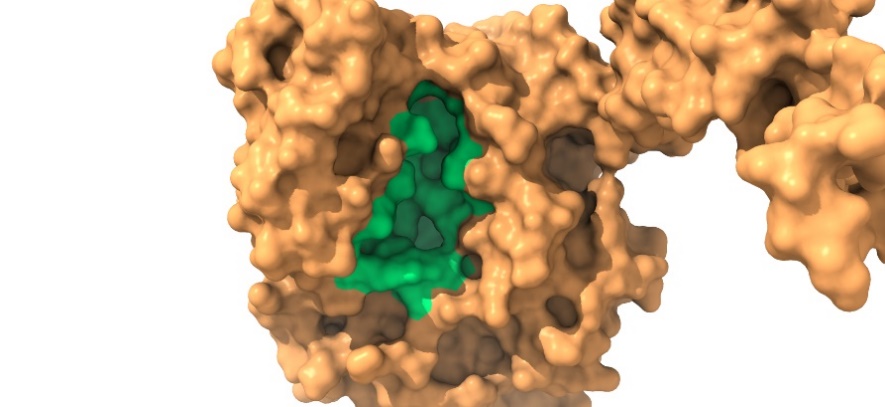 | **B.**  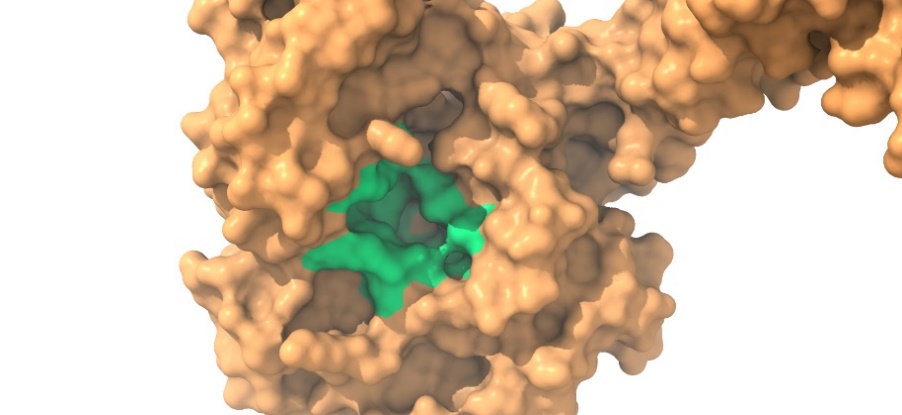 |
| --- | --- |
| **C.**  **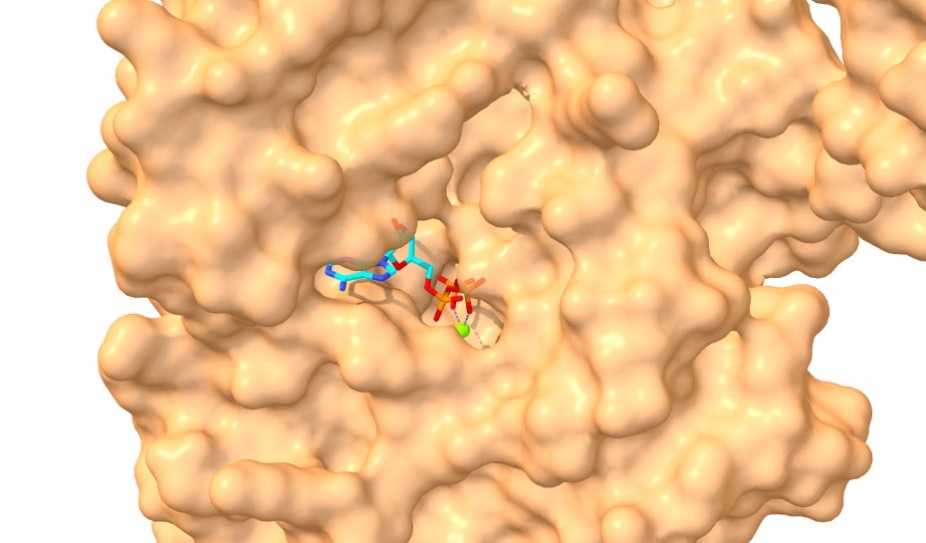** | |
| **Figure S7**. Visualization of P2Rank predicted docking pocket (green) of **A**. *Pseudomonas aeruginosa* and **B**. *Staphylococcus aureus* DnaK proteins via ChimeraX. **C.** ATP-bound (cyan coloured) structure of E. coli DnaK protein (brown) deposited in PDB (PDB ID: 4JNE, Chain: A) visualized for comparison to the predicted pocket. | |

**Docking Method Validation via Redocking**

| **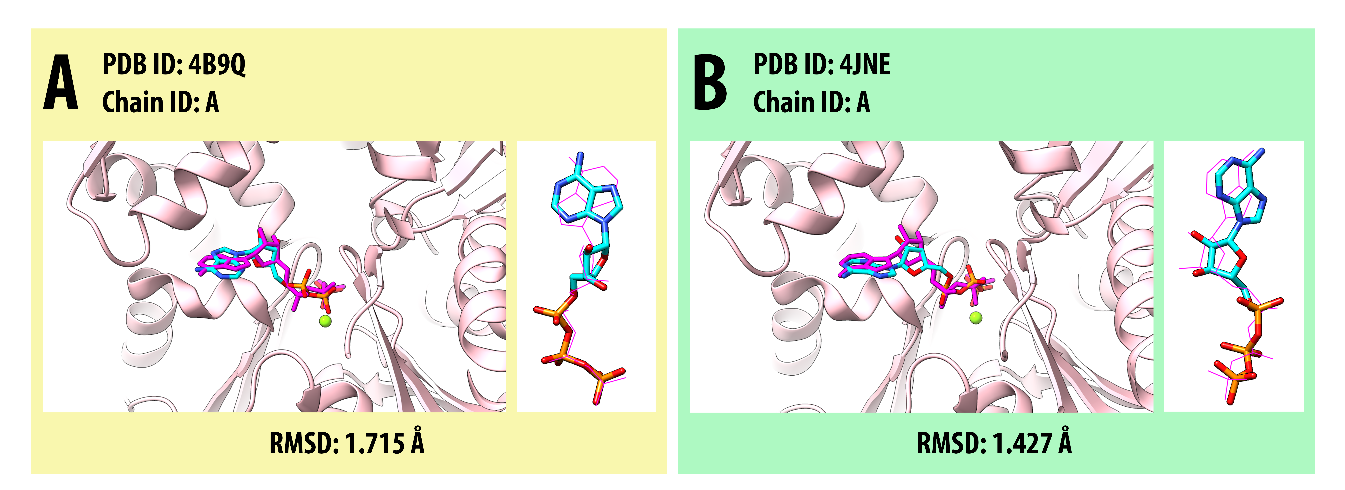** |
| --- |
| **Figure S8**. Validation of molecular docking methodology via redocking of reference ligands with crystallization proven binding pose. ATP-bound 3D models of *E. coli* DnaK protein available in PDB (**A.** PDB ID: 4B9Q, and **B.** PDB ID: 4JNE) were retrieved for validation, in accordance with the research objective of screening for ATP competitive inhibitors in DnaK protein. Highest ranked binding pose of 4B9Q and 4JNE were shown in cyan, with respective binding energies of -10.52 kcal/mol and -10.69 kcal/mol, as well as RMSD values of 1.715 Å and 1.427 Å with respect to reference binding poses (magenta). |

**MD Simulation: Energy Minimization and Equilibration**

| **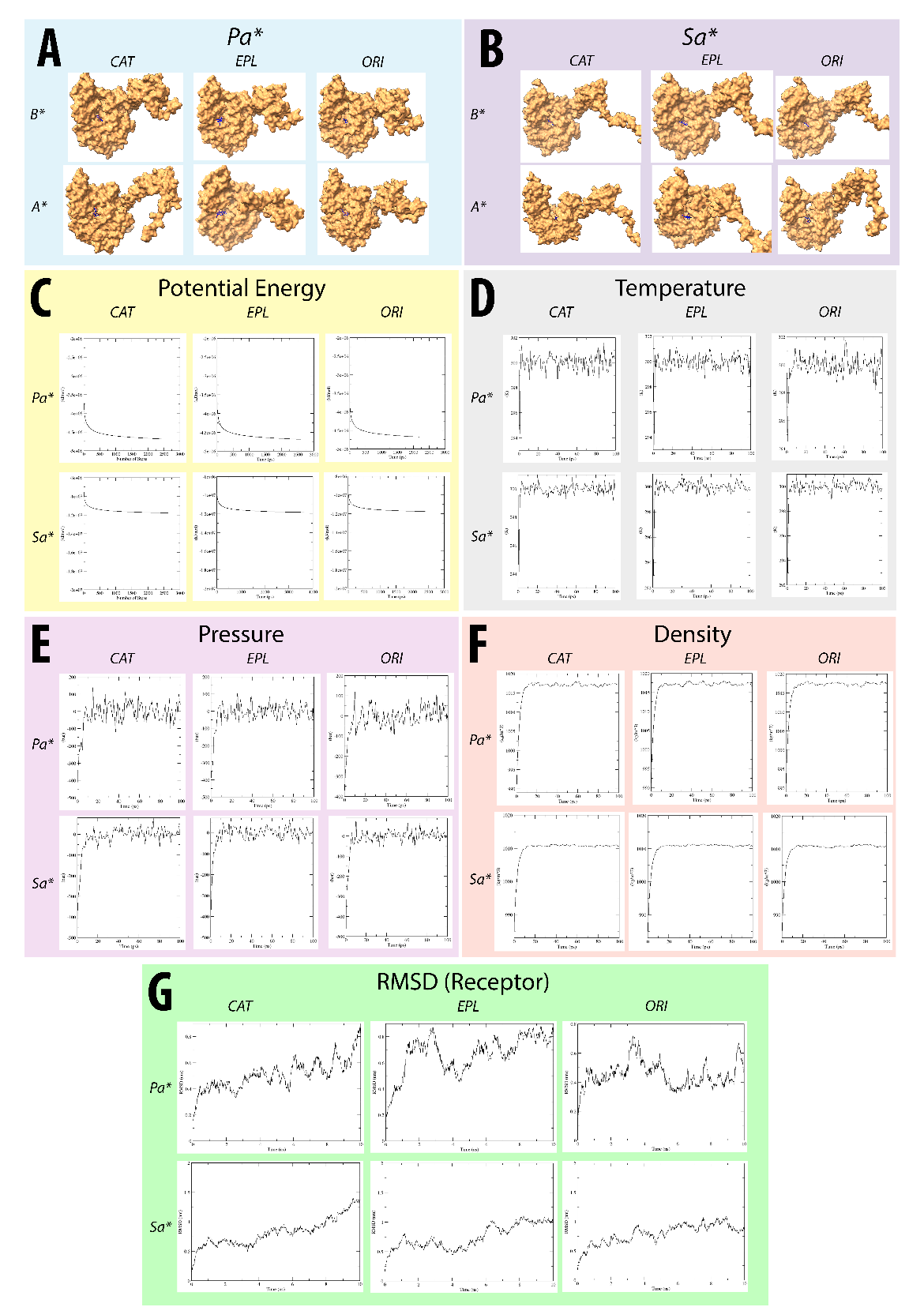** |
| --- |
| **Figure S9**. Energy minimization, NVT/NPT equilibration, and MD simulation for DnaK-ligand complexes. **A.** Full view of DnaK-ligand complexes before and after simulation. Convergence of energies, for instance, **B**. potential energies during steepest descent energy minimization, **C**. temperature during NVT equilibration, as well as **D.** pressure and **E**. densities during NPT equilibration, were visualized to ensure the stabilization of systems before simulations. **G**. Total RMSD of DnaK protein over the course of MD were also computed. *Note*. B* - Before MD, A* - After MD, Pa* - *P. aeruginosa*, Sa* - *S. aureus*, CAT – Catechin, EPL – Eplerenone, ORI – Oritin-4-beta-ol. |
